# Alkbh8 Regulates Selenocysteine-Protein Expression to Protect against Reactive Oxygen Species Damage

**DOI:** 10.1371/journal.pone.0131335

**Published:** 2015-07-06

**Authors:** Lauren Endres, Ulrike Begley, Ryan Clark, Chen Gu, Agnieszka Dziergowska, Andrzej Małkiewicz, J. Andres Melendez, Peter C. Dedon, Thomas J. Begley

**Affiliations:** 1 Colleges of Nanoscale Science and Engineering, SUNY Polytechnic Institute, Albany, New York 12203, United States of America; 2 RNA Institute and Cancer Research Center, University at Albany, State University of New York, Albany, New York 12222, United States of America; 3 Department of Biological Engineering, Massachusetts Institute of Technology, Cambridge, Massachusetts 02139, United States of America; 4 Institute of Organic Chemistry, Lodz University of Technology, Lodz, Poland; 5 Singapore-MIT Alliance for Research and Technology, CREATE, Singapore, Singapore; 6 Center for Environmental Health Sciences, Massachusetts Institute of Technology, Cambridge, Massachusetts 02139, United States of America; National Institute of Environmental Health Sciences, UNITED STATES

## Abstract

Environmental and metabolic sources of reactive oxygen species (ROS) can damage DNA, proteins and lipids to promote disease. Regulation of gene expression can prevent this damage and can include increased transcription, translation and post translational modification. Cellular responses to ROS play important roles in disease prevention, with deficiencies linked to cancer, neurodegeneration and ageing. Here we detail basal and damage-induced translational regulation of a group of oxidative-stress response enzymes by the tRNA methyltransferase Alkbh8. Using a new gene targeted knockout mouse cell system, we show that *Alkbh8^-/-^* embryonic fibroblasts (MEFs) display elevated ROS levels, increased DNA and lipid damage and hallmarks of cellular stress. We demonstrate that Alkbh8 is induced in response to ROS and is required for the efficient expression of selenocysteine-containing ROS detoxification enzymes belonging to the glutathione peroxidase (Gpx1, Gpx3, Gpx6 and likely Gpx4) and thioredoxin reductase (TrxR1) families. We also show that, in response to oxidative stress, the tRNA modification 5-methoxycarbonylmethyl-2′-O-methyluridine (mcm^5^Um) increases in normal MEFs to drive the expression of ROS detoxification enzymes, with this damage-induced reprogramming of tRNA and stop-codon recoding corrupted in *Alkbh8^-/-^* MEFS. These studies define Alkbh8 and tRNA modifications as central regulators of cellular oxidative stress responses in mammalian systems. In addition they highlight a new animal model for use in environmental and cancer studies and link translational regulation to the prevention of DNA and lipid damage.

## Introduction

Mammalian alkylation repair homolog 8 (Alkbh8) belongs to a family of nine related proteins, Alkbh1-8 and fat mass and obesity associated (FTO) that all share a conserved 2-oxoglutarate-Fe(II) oxygenase domain (2OG-Fe(II))[1–3]. This domain is structurally homologous to that of the bacterial AlkB protein, the likely ancestral protein. The bacterial 2OG-Fe(II) domain associated with *E*. *coli* AlkB is known to catalyze the oxidative demethylation of 1-methyladenine (1-meA) and 3-methylcytosine (3-meC) bases in DNA and RNA, a function that has also been attributed to mammalian Alkbh1, Alkbh2 and Alkbh3 *in vitro* and to Alkbh2 *in vivo* [4–9]. Alkbh5 is an RNA demethylase that works on N^6^-methyladenosine found in mRNA to regulate the expression of these methylated transcripts[10]. Spermatogenesis is noticeably defective in *Alkbh5* ^*-/-*^ mice, with genomic studies suggesting a link between Alkbh5 and p53 [10]. The function of Alkbh family members also extends beyond that of nucleic acid modification and repair, as there is evidence to support that Alkbh1 and 4 are involved in regulating gene expression through histone demethylation and interactions with regulators of transcription, respectively [11–13].

Alkbh8 is unique among the Alkbh8 family members because in addition to the 2OG-Fe(II) domain it contains a methyltransferase domain as well as an RNA binding motif. The methyltransferase domain of mouse and human Alkbh8 is homologous to *S*. *cerevisiae* tRNA methyltransferase 9 (Trm9) and both methylate RNA to complete the formation 5-methoxycarbonylmethyluridine (mcm^5^U) and 5-methoxycarbonylmethyl-2-thiouridine (mcm^5^s^2^U) at the wobble position of specific tRNAs for arginine and glutamic acid (tRNA^UCU-ARG^ & tRNA^UUC-GLU^) [14–16]. Defects in Alkbh8 also result in decreased 5-methoxycarbonylmethyl-2′-O-methyluridine (mcm^5^Um), which could be due to an enzyme deficiency or the need for mcm^5^U as a substrate [15]. Previously, we demonstrated links between protein translation, tRNA modifications and stress responses that include ROS- and DNA damage (DDR) responses: we have shown that a deficiency in Trm4-catalyzed m^5^C wobble base modifications corrupt the cellular response to ROS and DNA damaging agents [17], and have also established that Trm9-deficient yeast cells are sensitive to killing by DNA damaging agents [18–20]. Mechanistically, Trm9-dependent wobble uridine modifications optimize the translation of the DDR proteins ribonucleotide reductase 1 and 3 through enhanced codon-biased translation (Rnr1 and Rnr3) [18, 20]. Further, we have demonstrated that the Trm9 dependent modification mcm^5^U is increased 2-fold in S-phase during DNA damage conditions, with increases also occurring in response to methyl methanesulfonate (MMS), *N*-methyl-*N'*-nitro-*N*-nitrosoguanidine (MNNG), ethyl methanesulfonate (EMS) and isopropyl methanesulfonate (IMS) exposures [20](Chan et al., submitted).

There are some notable differences between yeast Trm9 and mammalian Alkbh8 proteins. The 2OG-Fe(II) oxygenase domain is only found on Alkbh8 and is known to directly hydroxylate the *S*-diastereomer of 5-methoxycarbonylhydroxymethyluridine (mchm^5^U) in tRNA^UCC-GLY^ from the wobble mcm^5^U precursor [21]. Alkbh8 activity is required for mcm^5^U and mcm^5^Um modifications at the wobble position of selenocysteine tRNA (tRNA^UGA-SEC^) [15] as Alkbh8 generates the substrate (mcm^5^U) for the yet to be identified activity that modifies the ribose sugar to make mcm^5^Um. Selenocysteine does not have a designated triplet codon; therefore, amino acid decoding is unconventional and requires recoding of the UGA stop for incorporation [22, 23]. Budding yeast does not have selenocysteine charged tRNA or have mcm^5^Um modified tRNA. In mammalian systems an essential component for UGA stop codon recoding is methylation (mcm^5^U and mcm^5^Um) at the wobble uridine of the tRNA^UGA-SEC^ [15, 24–28], with some supporting studies using tRNAs altered for two to three modifications in the anticodon. There are over 23 selenocysteine containing proteins in mammals that are potentially regulated by mcm^5^U-based modifications linked to Alkbh8 activity, and they include glutathione peroxidases (Gpx) and thioredoxin reductases (TrxRs) [27, 29, 30]. Comprising one of the most important antioxidant enzyme systems in humans, Gpx proteins catalyze the hydrogen atom transfer from reduced glutathione to electrophilic centers, effectively detoxifying potentially harmful cellular reactive oxygen compounds like H_2_O_2_ and peroxidized lipids [31]. Thioredoxin reductases (TrxRs) comprise another family of selenocysteine-containing antioxidant proteins. NADPH acts as the electron donor for TrxRs, which use FAD and the active site selenocysteine in a series of redox reactions that culminate in reduced thioredoxin (Trx) [32], with Trx being an antioxidant that donates electrons to peroxidases and ribonucleotide reductases (RNR) [33].

Due to the homology between the stress-important methyltransferase domains of yeast Trm9 and mammalian Alkbh8, we were motivated to investigate stress-phenotypes and stress-responses in Alkbh8 deficient mouse cell lines. To this end, we used Alkbh8 gene-targeted mouse embryonic cells to generate a new Alkbh8 deficient (*Alkbh8* ^*-/-*^) mouse. In our current study we find that, when grown under basal non-stressed cell culture conditions, murine embryonic fibroblasts (MEFs) derived from *Alkbh8* ^*-/-*^ mice have a slow growth phenotype, and also have increased DNA strand breaks and an activated DNA damage response, relative to their wild type (wt) counterparts. A high degree of *Alkbh8* ^*-/-*^ MEF sensitivity was observed in response to DNA damaging agents that induce oxidative stress. Consistent with this sensitivity phenotype, we found that *Alkbh8* ^*-/-*^ MEFs have increased levels of intracellular reactive oxygen species (ROS), lipid peroxidation products and a transcript expression signature indicative of oxidative stress. To mechanistically link the ROS and DNA damage phenotypes of the *Alkbh8* ^*-/-*^ MEFs, we have demonstrated that *Alkbh8* ^*-/-*^ MEFs have decreased Gpx1, Gpx3, Gpx6 and TrxR1 protein expression, an effect that was pronounced after H_2_O_2_ exposure. Further, we show that Alkbh8 levels are increased in response to ROS to help drive the increased expression of ROS detoxification activities. Lastly, we show that stop codon recoding and the mcm^5^Um modification are increased in response to H_2_O_2_ exposure in wt MEF’s, with both being significantly decreased in our *Alkbh8* ^*-/-*^ MEFS. Our results support a model in which Alkbh8 regulates the cellular redox state under both basal and increased ROS conditions, via modulation of stop codon recoding, mcm^5^Um and selenocysteine protein expression. Importantly we demonstrate that Alkbh8 regulates an ROS detoxification network reliant on increased stop codon recoding and mcm^5^Um tRNA modifications. The work herein establishes a role for Alkbh8 in the oxidative stress response and the prevention of DNA damage, and suggests that Alkbh8 may be linked to pathologies.

## Materials and Methods

### Alkbh8 deficient (Alkbh8^-/-^) mouse generation, genotyping and MEF isolation

The animal model was cared for under approval from the University at Albany Institutional Animal Care and Use Committee (IACUC) which specifically approved this study; #11–053. The Alkbh8 gene was targeted in the parental E14Tg2a.4 129P2 embryonic stem (ES) cell line using an insertion mutagenesis approach in which a vector containing a splice acceptor sequence upstream of a ß-geo cassette (ß-galactoside/neomycin phosphotransferase fusion) was inserted into intron 7 at chromosome position 9:3349468–3359589, creating a gene fusion Alkbh8-ß-geo truncated transcript [34]. This ES cell line was obtained from the Mutant Mouse Regional Resource Center as BayGenomics Alkbh8^Gt(RRY122)Byg^, and was used for injection into C57BL/6 blastocysts to create chimeras, followed by breeding to produce Alkbh8^-/-^ mice. Multiplexed qRT-PCR and relative cycle threshold analysis (ΔΔCt) on genomic DNA derived from tail biopsies was used to determine animal zygosity with TaqMan oligonucleotides specific for neomycin (Neo, target allele) and T-cell receptor delta (Tcrd, internal positive control). Neo forward primer: 5'-CCA TTC GAC CAC CAA GCG-3', Neo reverse primer: 5'-AAG ACC GGC TTC CAT CCG-3', Neo probe: 5'-FAM AAC ATC GCA TCG AGC GAG CAC GT TAMRA-3', Tcrd forward primer: 5'-CAG ACT GGT TAT CTG CAA AGC AA-3', Tcrd reverse primer: 5'-TCT ATG CCA GTT CCA AAA AAC ATC-3', and Tcrd probe: 5'-VIC ATT ATA ACG TGC TCC TGG GAC ACC C TAMRA-3'. Secondary confirmation of the truncated Alkbh8 mRNA transcript was achieved by qRT-PCR and absolute quantity analysis with a Taqman Gene Expression assay targeting exons 9–10 (i.e. 3' of the insertion site), using the mouse Alkbh8 open reading frame as a standard. Thermo-cycling conditions on an Applied Biosystems StepOnePlus RT-PCR machine were as follows: 50°C for 2 min, 95°C for 10 min, and 40x (95°C for 15 s and 60°C for 1 min). Primary murine embryonic fibroblasts (MEFs) were isolated from individual *Alkbh8* ^*-/-*^ and wild type littermate 12.5 day embryos, and were maintained in Dulbecco's modified Eagle's media supplemented with 10% heat inactivated fetal bovine serum (Sigma, MO), GIBCO non-essential amino acids, 100 Units/mL penicillin and 100 micrograms/mL streptomycin, at 37°C and 5% CO_2_. Immortalized MEFs were established under the same culturing conditions using a 3-day transfer regime [35].

### Cell viability, cell cycle and apoptosis measurements

Cell viability was determined by crystal violet staining (0.2% w/v in 2% ethanol) and Trypan blue or propidium iodide exclusion (GIBCO-Invitrogen, CA). For cell cycle analysis, MEFs were fixed in ice in 70% ethanol, washed with 1% BSA-PBS, incubated with 1 mg/mL RNase A for 10 min at 37°C, and then resuspended in PBS containing 0.1 mg/mL propidium iodide. Acquisition and analysis of DNA content was by flow cytometry using an LSR II cytometer (Becton Dickinson, NJ) and FlowJo software (Tree Star, OR). Apoptotic cell populations were determined by TUNEL assay (Roche, IN) according to the manufacturer’s recommendations with minor changes: cells were first trypsinized to make a single cell population, which was then fixed, permeabilized and labeled with dUTP-conjugated to FITC. The percentage of TUNEL positive apoptotic cells was determined by flow cytometry enumeration of fluorescent cells (FITC-A: 570–620 nm).

### Microarray analysis

Total RNA was isolated from passage 3 primary mouse embryonic fibroblasts (MEFs) using Trizol reagent (Ambion, CA) according to the manufacturer's protocol. cDNA synthesis was performed using Ovation RNA Amplification System v2 kit (Nugen, CA) to produce biotin-labeled probes representing either *Alkbh8* ^*-/-*^ or wild type RNA populations, which were then hybridized to the GeneChip Mouse Gene 1.0 ST Array (Affymetrix, CA), one RNA sample per array. Signal intensity analyses were performed with GeneSpring GX software (Agilent Technologies, CA) with a corrected p-value cut-off of 0.05. Statistically significant differences in mean fluorescence intensities were determined using the unpaired Student's t-test.

### Quantitative real-time PCR (qRT-PCR), Western blot analysis and Gpx activity assay

A pathway-focused RT^2^ Profiler PCR array (PAMM-069 customized to include *Gsto2*, *Adh7*, *Sod3* and *Gstm7*) was used to analyze oxidative stress gene expression and first strand cDNA synthesis and real-time PCR conditions were performed in accordance with the manufacturer’s instructions (SABiosciences/Qiagen, MD). *PERP*, *Actin* and *Alkbh8* gene expression were detected using Taqman Gene Expression assays and all qRT-PCR was carried out using an Applied Biosystems StepOne Plus detection system, with each sample tested in triplicate. Commercial antibodies used in this study were as follows: total p53 and GAPDH (Calbiochem, MA), Gpx1 and Alkbh8 (Santa Cruz Biotechnology, CA), Gpx3 and 6 (Abcam, MA) and Trx1 and 2 (Pierce, IL). Gpx activity was measured in whole cell lysates using a Gpx assay kit (SIGMA, MO), according to the manufacturer's specifications and using a total of 200 μg of protein in 250 μL for activity in units/mL.

### Nucleofection and Stop codon recoding reporter assay

pcDNA3.1-EV, pcDNA3.1-Alkbh8 and pCMV-Script-DualLuc-Gpx-SECIS [15] were introduced into wt and *Alkbh8* ^*-/-*^ MEFs using a 4D-Nucleofector System (Lonza, Basel, Switzerland), according to the manufacturer's protocol, after optimizing conditions for primary MEFs (*i*.*e*. solution P2, program EN-150). Immunoblot analysis was performed twenty-four hours post nucleofection, as described above. Under conditions of stop codon recoding the UGA stop codon is "read-through" resulting in the expression of the firefly enzyme. Twenty-four hours post-nucleofection, MEFs were treated with 1.2 mM H_2_O_2_, or left untreated. Twenty hours post-H_2_O_2_ exposure, a dual-luciferase reporter assay (Promega) was performed according to the maufacturer's protocol, and *Renilla* and firefly luciferase activities were measured using a Tecan M200 plate reader with monochromator-based wavelength selection for luminescence.

### ROS detection, Comet assay and γ-H2AX foci analysis

Fresh 2’,7’-dichlorofluorescin (DCFDA, Molecular Probes-Invitrogen, CA) was dissolved in DMSO on the day of the experiment to a concentration of 10 mg/mL, which was added directly to the cell culture media to a final concentration of 10 μg/mL for 30 min at 37°C. Cells were then analyzed by flow cytometry using an LSRII (BD Biosciences, CA) or Image Stream ISX100 (Amnis, WA) cell analyzers, and the level of reactive oxygen species was measured based on the emission of oxidized DCFDA dye (517–527 nm). For ROS induction, cells were treated with an acute dose of hydrogen peroxide at a concentration of 1.2 millimolar for 30 min. For ROS mitigation, cells were pretreated with 20 mM N-acetyl cysteine (NAC) for 1 h, rinsed twice with Phosphate-buffered saline, followed by ROS induction with H_2_O_2_.

An alkaline comet assay was performed using the Trevigen (MD) CometAssay kit with a total of 5 x 10^5^ cells suspended in low-melt agarose. After cell lysis and electrophoresis, nuclei were stained with SYBER Green 1 and Comet tails were visualized by epifluorescence microscopy (521 nm). Quantitative image analysis of Comet tails was performed using Tritek CometScore software and is presented as the percentage of DNA in the Comet tail. Single-cell populations were prepared for staining with FITC-conjugated anti-phospho(Ser139)-H2AX antibody (**γ**-H2AX-FTIC, Millipore) using the same procedure as described for TUNEL assay. Cells (2x10^6^) were incubated with 2 μg of **γ**-H2AX-FITC, or anti-mouse IgG-FITC as a control for back ground staining, on ice for 30 min. Cell pellets were washed twice with Tris-buffered saline containing 0.1% Triton-X-100, followed by final resuspension in Phosphate-buffered saline containing 2% Fetal Bovine Serum and 5 μM DRAQ5 (Cell Signaling Technology). Quantitative image analysis of nuclear (DRAQ5: > 655 nm) and **γ**-H2AX foci (FITC: 570–620 nm) was performed using an ImageStreamX cytometer (Amnis) and the Spot Count analysis software program.

### Lipid peroxidation (LPO) analysis

*Alkbh8* ^*-/-*^ and wt MEF cells were cultured in 6-well format and treated with 1.2 mM H_2_O_2_ in serum-free media for 1 h. After treatment media was replaced with complete growth media and incubated for 48 h in a humidified CO_2_-incubator. Cells were harvested and lysed in RIPA buffer (50 mM Tris-HCl, pH 7.4, with 150 mM sodium chloride, 1% TritonX-100, 0.5% sodium deoxycholate, and 0.1% sodium dodecyl sulfate) supplemented with an EDTA-free protein inhibitor cocktail (Roche Diagnostics). Extracted protein was normalized using Bradford (BioRad) analysis according to manufacturer’s instruction. LPO was analyzed using the 8-Isoprostane EIA assay kit from Cayman Chemical Company (Ann Arbor, MI, USA). The 96-well assay plate was set up and run as suggested by the manufacturer, using an eight point standard curve run in duplicate. Protein samples were diluted in EIA buffer (supplied by the manufacturer) to a final concentration of 0.250 μg per well. Data reported comes from biological triplicates, with each sample assayed in duplicate. The 8-isoprostane sample concentrations from the mean absorbance were interpolated from a sigmoid standard curve (4 parameter logistic nonlinear regression) using Graph Pad software. A Student t-test was used to identify significant differences between groups at p < 0.05.

### tRNA modification analysis

The levels of three AlkBH8-related ribonucleosides in tRNA, mcm^5^s^2^U, mcm^5^U, and mcm^5^Um, were quantified in triplicate for each experimental condition using a protocol slightly modified from Su et al. (2014). tRNA was isolated as the major component (>85%) of small RNA isolated from mouse embryonic fibroblast (MEF) cells and livers using the miRNeasy Mini Kit (Qiagen). The tRNA (2 μg) was hydrolyzed and dephosphorylated in the presence of antioxidants and deaminase inhibitors and 20 nM [^15^N]_5_-deoxyadenosine to serve as an internal standard, as described in detail elsewhere [19, 36]. Following the enzymatic digestion, proteins were subsequently removed by filtration (YM-10, Pall). For MEF cell samples, the ribonucleosides in the filtrate were resolved by HPLC (Thermo Scientific Hypersil GOLD aQ reversed-phase column, 150 × 2.1 mm, 3 μm particle size) with a 0–75% gradient of acetonitrile in water containing 0.1% formic acid at a flow rate of 0.4 mL/min at 36°C: 0–6 min, 0%; 6–7.65 min, 0–1%; 7.65–9.35 min, 1–6%; 9.35–10 min, 6%; 10–12 min, 6–50%; 12–14 min, 50–75%; 14–17 min, 75%. The HPLC system was directly coupled to an Agilent 6430 QqQ mass spectrometer with an ESI source operating in positive ion mode: gas temperature, 350°C; N_2_ gas flow, 10 L/min; nebulizer pressure, 40 psi; capillary voltage, 3800 V. The mass spectrometer was set to run at the segmented Multiple Reaction Monitoring (MRM) mode (unit resolution in both Q1 and Q3, Delta EMV = 400 V), measuring a list of mass transitions. For both mcm^5^U and mcm^5^Um, the corresponding MS signal was assigned by comparing the retention time of the signal in one or two characteristic MRM transitions to that of a synthetic standard; the peak attributed to mcm^5^s^2^U in two characteristic MRM transitions was verified by its complete absence in the ribonucleosides hydrolyzed from the tRNA of a *S*. *cerevisiae trm9*Δ and its presence in the wild-type *S*. *cerevisiae* By4741 [19]. The quantity of each ribonucleoside was calculated from the area under the curve of the designated peak of the strongest MRM transition for each ribonucleoside (mcm^5^U: 317 → 185; mcm^5^Um: 331 → 185; mcm^5^S^2^U: 333 → 201). This value was then normalized to account for sample-to-sample variation in tRNA concentration by dividing the MS peak area by the total number of moles of canonical ribonucleosides (i.e., cytidine, uridine, adenosine, guanosine) present in each sample. Canonical ribonucleosides were quantified from UV chromatograms obtained with an in-line UV detector during the LC-MS/MS run, using published extinction coefficients [37]. For mouse liver samples, post enzymatic hydrolysis the ribonucleosides in the filtrate were resolved using a slightly modified HPLC method (Phenomenex Synergy Fusion RP column, 100 × 2 mm, 2.5 μm particle size, 100 Å pore size), with the following gradient in 5 mM aqueous NH_4_OAc (pH = 5.3)/Acetonitrile at a flow rate of 0.35 ml/min at 35°C: 0–1 min, 0%; 1–10 min, 0–10%; 10–14 min, 10–40%; 14–15 min, 40–80%; 15–16 min, 80–0%; 16–20 min, 0%; The LC-coupled Agilent 6430 QqQ mass spectrometer with an ESI source was set to operate in positive ion mode: gas temperature, 350°C; N_2_ gas flow, 10 L/min; nebulizer pressure, 40 psi; capillary voltage, 3500 V. The mass spectrometer was configured to run at the Dynamic Multiple Reaction Monitoring (DMRM) mode (unit resolution in both Q1 and Q3, Delta EMV = 400 V), measuring a list of mass transitions including mcm^5^U (317.2 → 185, Fragmentor = 90V, CE = 8 eV, RT = 6.5 ± 1.2 min), mcm^5^Um (331.2 → 153, Fragmentor = 90V, CE = 8 eV, RT = 9.0 ± 1.2 min), and mcm^5^S^2^U (333 → 201, Fragmentor = 100V, CE = 10 eV, RT = 9.1 ± 1.2 min). The quantity of each ribonucleoside was calculated from the area under the curve of the designated peak of the DMRM transition for each ribonucleoside and then normalized against the canonical ribonucleosides as mentioned above.

## Results

### Alkbh8^-/-^ mouse embryonic fibroblast cells (MEFs) demonstrate slow growth and increased cell death

**Fig 1:**
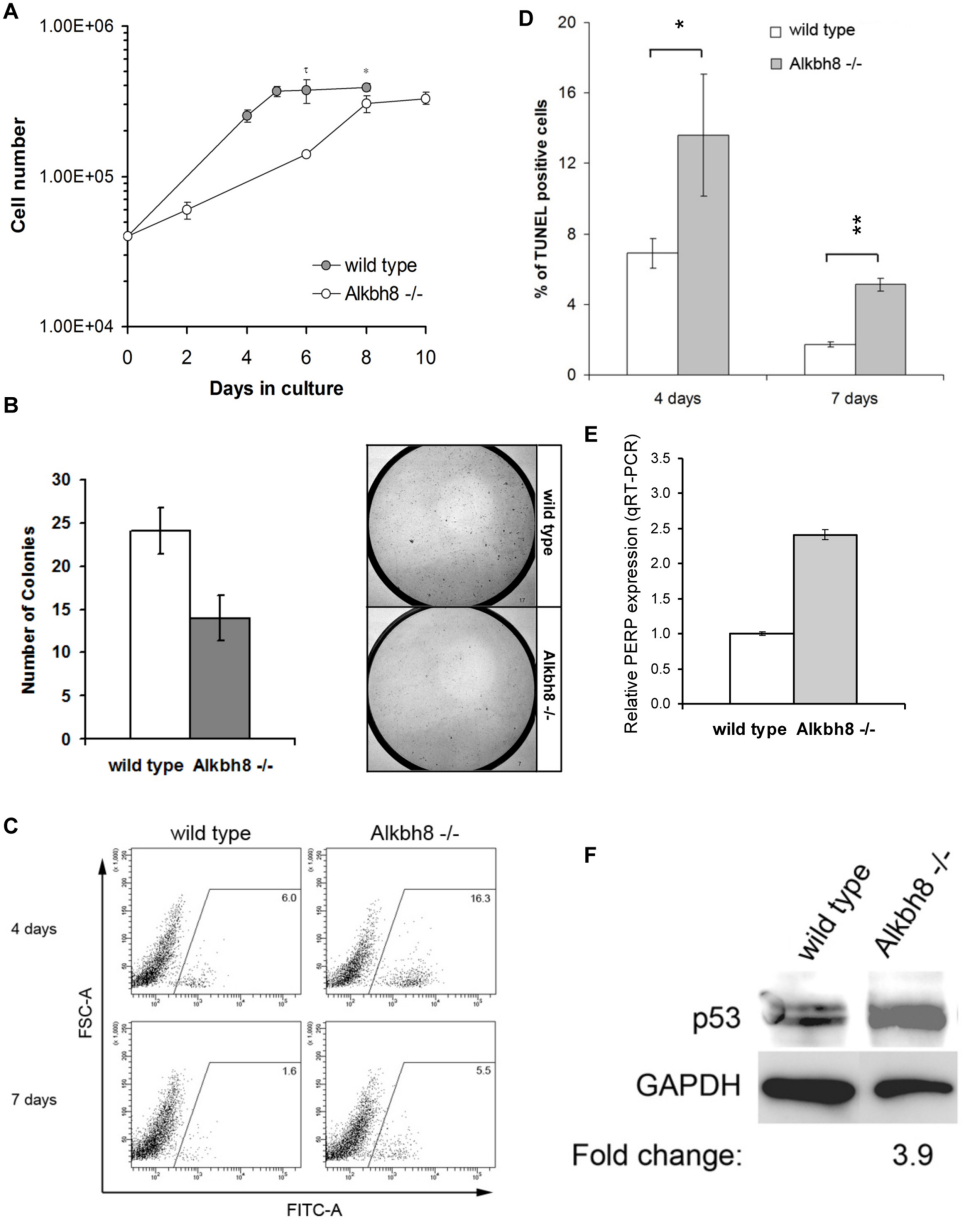
*Alkbh8* ^*-/-*^ MEFs have slow growth and increased apoptosis phenotypes. **A)** 4 x 10^4^ wt or *Alkbh8* ^*-/-*^ MEFs were seeded per 9.6 cm^2^ well and viable (trypan blue negative) cells were counted over a period of 12 days. Error bars represent standard deviation (±STDV, n = 3) and significant differences in growth was determined by Student t-test (^**τ**^p < 0.005, *p < 0.05). **B)** 1 x 10^4^ cells were seeded per 58.1 cm^2^ dish, stained with crystal violet after 2 weeks in culture and colonies with a diameter > 2 mm were counted. Representative pictures of crystal violet stained cells. **C-D)** MEFs were allowed to grow to confluence and 2.5 x 10^5^ cells were plated per 58.1 cm^2^ dish. Cells were fixed and assessed for apoptosis by TUNEL assay using a dUTP-FITC conjugate, 4 and 7 days after plating. Apoptotic cells were enumerated by flow cytometry and appear in the FITC-A channel. The percentage of TUNEL-positive cells is shown as the mean ± standard deviation (n = 3, right panel, *p < 0.05, **p < 0.001). **E-F)** RNA or whole protein extracts were prepared from sub-confluent cultures of wt and *Alkbh8* ^*-/-*^ MEFs and assessed for **(E)** PERP gene expression by qRT-PCR, in which each sample was normalized to internal Gapdh levels and **(F)** p53 protein expression by Western blotting

Mice were made Alkbh8 deficient using an insertional mutagenic approach that introduced a "gene trap" into intron 7 [34]. This insertion creates a fusion transcript with a premature stop codon upstream of coding exons 8–11 and thus truncates not only the entire methyltransferase domain but also a portion of the 2OG-Fe(II) (S1 Fig). In culturing *Alkbh8* ^*-/-*^ MEFs, it became apparent that they grew more slowly relative to their wt counterparts. As a first step in phenotype characterization studies, the growth and colony forming potential of *Alkbh8* ^*-/-*^ MEFs were assessed and compared to wt MEFs. During a 10-day period of culture, the *Alkbh8* ^*-/-*^ MEFs grew approximately 2-times slower than did the wt MEFs (Fig 1A). Further, *Alkbh8* ^*-/-*^ MEFs plated at low density formed half the number of colonies than did the wt MEFs after two weeks of culturing (Fig 1B). The slow growth and diminished colony forming phenotypes suggest that *Alkbh8* ^*-/-*^ were surviving under stress, a trait common to other stress response and DNA repair-deficient cells [38].

To further characterize the growth phenotypes of *Alkbh8* ^-/-^ MEFs in comparison to wt MEFs, we measured apoptosis and analyzed cell cycle profiles. We used the TUNEL assay combined with flow cytometry to obtain a quantitative measurement of apoptosis in *Alkbh8* ^*-/-*^ and wt MEFs. Both cells were plated at low density and TUNEL positive cells were enumerated 4 and 7 days post-plating. The percentage of apoptotic cells was consistently higher for *Alkbh8* ^*-/-*^ MEFs compared to wt MEFs and both percentages decreased with time, which is likely due to the cultures reaching confluence (Fig 1C and 1D). Cells with a higher apoptotic index are also expected to express cell death effectors. Consistent with this reasoning, we observed an increase in the transcript level of PERP, a known p53 gene target and apoptotic effecter (Fig 1E), as well as elevated p53 protein levels in *Alkbh8* ^*-/-*^ MEFs (Fig 1F). Our cell cycle analysis did not reveal a significant difference in the percentage of replicating S-phase populations of wild type and *Alkbh8* ^*-/-*^ MEFs (S2 Fig). In light of these results, the slow growth phenotype of the *Alkbh8* ^*-/-*^ MEFs is likely accounted for by a higher incidence of apoptosis.

### Increased DNA damage and activation of DNA damage response in Alkbh8^-/-^ MEFs

**Fig 2:**
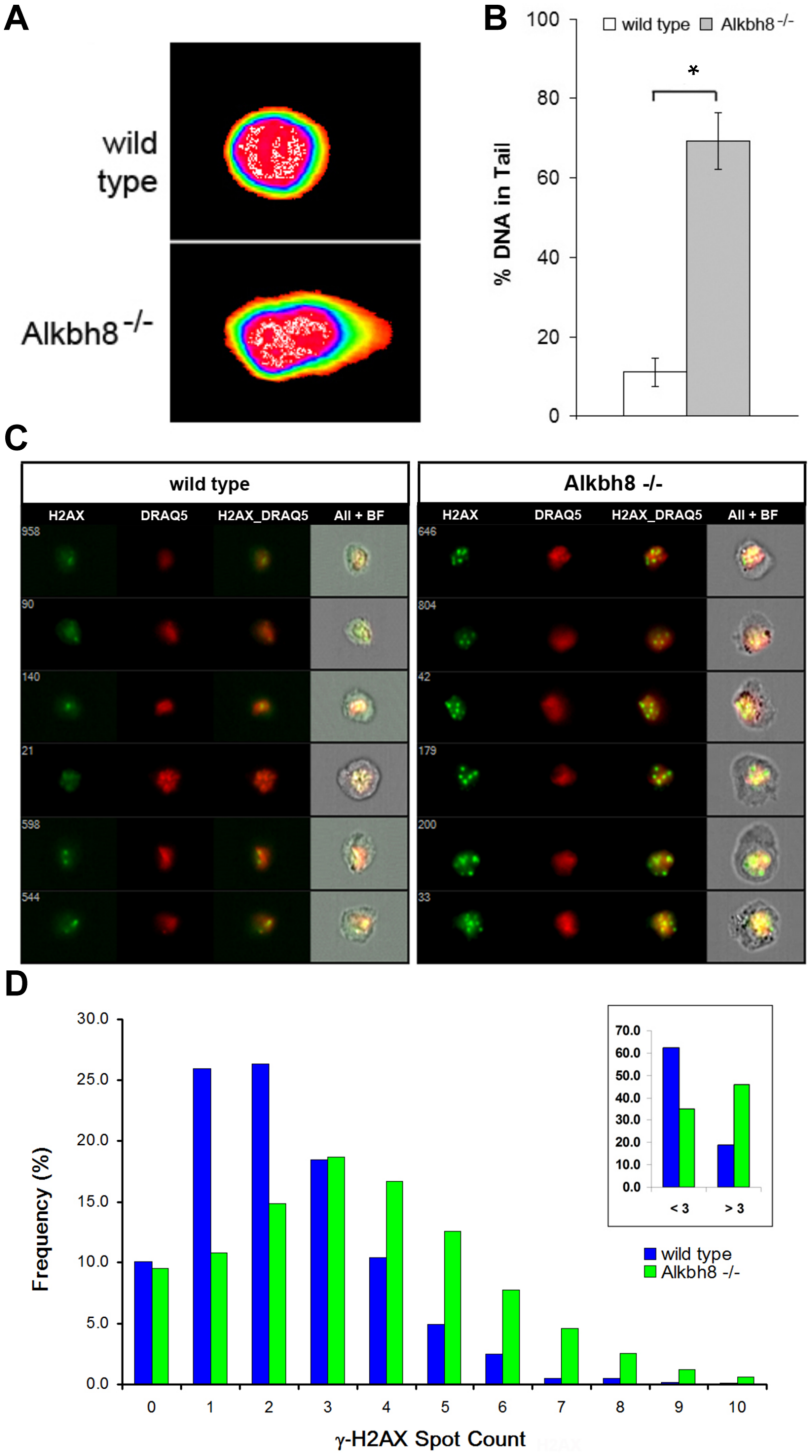
Increased DNA damage and activated DNA damage response in *Alkbh8* ^*-/-*^ MEFs. **A)** Comet assay of wt and *Alkbh8* ^*-/-*^ nuclei isolated from MEFs that were cultured as described above. **B)** Comet tails were quantified using CometScore and are shown as the mean % of DNA in the comet tail (±STDV, n = 3). Significant differences in the % of DNA in the comet tails of wt and *Alkbh8* ^*-/-*^ MEFs were determined by Student t-test *p < 0.001). **C)** Single cell suspensions were fixed and stained with a FITC conjugated antibody specific for **γ**-H2AX and the DRAQ5 nuclear stain followed by quantitative imaging with an ISX100 flow cytometer. Representative **γ**-H2AX foci images are shown for cell batches taken from the peak frequency of Spot Count distributions for wt and *Alkbh8* ^*-/-*^ sample runs. **D) γ**-H2AX foci were scored for intensity and number using the IDEAS Spot Count analysis program, presented as the percentage frequency of cells with 0–10 Spot Counts, and those with a Spot counts less than or greater than 3 (inset, n = 6).

Slow cell growth and an increased incidence of cell death are potential phenotypes for cells compromised for damage prevention, stress- and DDR-systems, defects that potentially manifest as unrepaired single or double strand DNA breaks. We used the Comet assay to determine whether *Alkbh8* ^*-/-*^ MEFs had DNA strand breaks. We found that under basal growth conditions, the nuclei derived from *Alkbh8* ^*-/-*^ MEFs had a much higher percentage of strand breaks compared to nuclei derived from wt MEFs (Fig 2A and 2B). An early event at sites of DSBs is the recruitment of the Mre11-Rad51-Nbs1 (MRN) complex, a process that promotes the phosphorylation of the histone protein H2AX on Ser129, (denoted **γ**-H2AX), which accumulates as distinct foci at sites of double strand breaks [39, 40]. We stained MEFs with a **γ**-H2AX specific antibody conjugated to FITC and used imaging flow cytometry to quantitate the number of **γ**-H2AX positive foci. In these experiments the number of cells containing 0–10 **γ**-H2AX positive foci (*i*.*e*., using a spot count algorithm) were calculated for both wt and *Alkbh8* ^*-/-*^ MEFs for 20,000 cells, with each spot count number represented as a total frequency. In general, the number of **γ**-H2AX foci per cell was increased in *Alkbh8* ^*-/-*^ MEFs, relative to wild type MEFs, under basal growth conditions. Specifically, 40% of the *Alkbh8* ^*-/-*^ MEF population had greater than three foci, while the wt population had only 18%. Our results demonstrate that an Alkbh8 deficiency leads to the activation of the DNA damage response, which is most likely due to an increase in DSBs (Fig 2C and 2D).

### Alkbh8 deficiency leads to increased sensitivity to DNA damaging agents, especially those agents that cause oxidative stress

**Fig 3:**
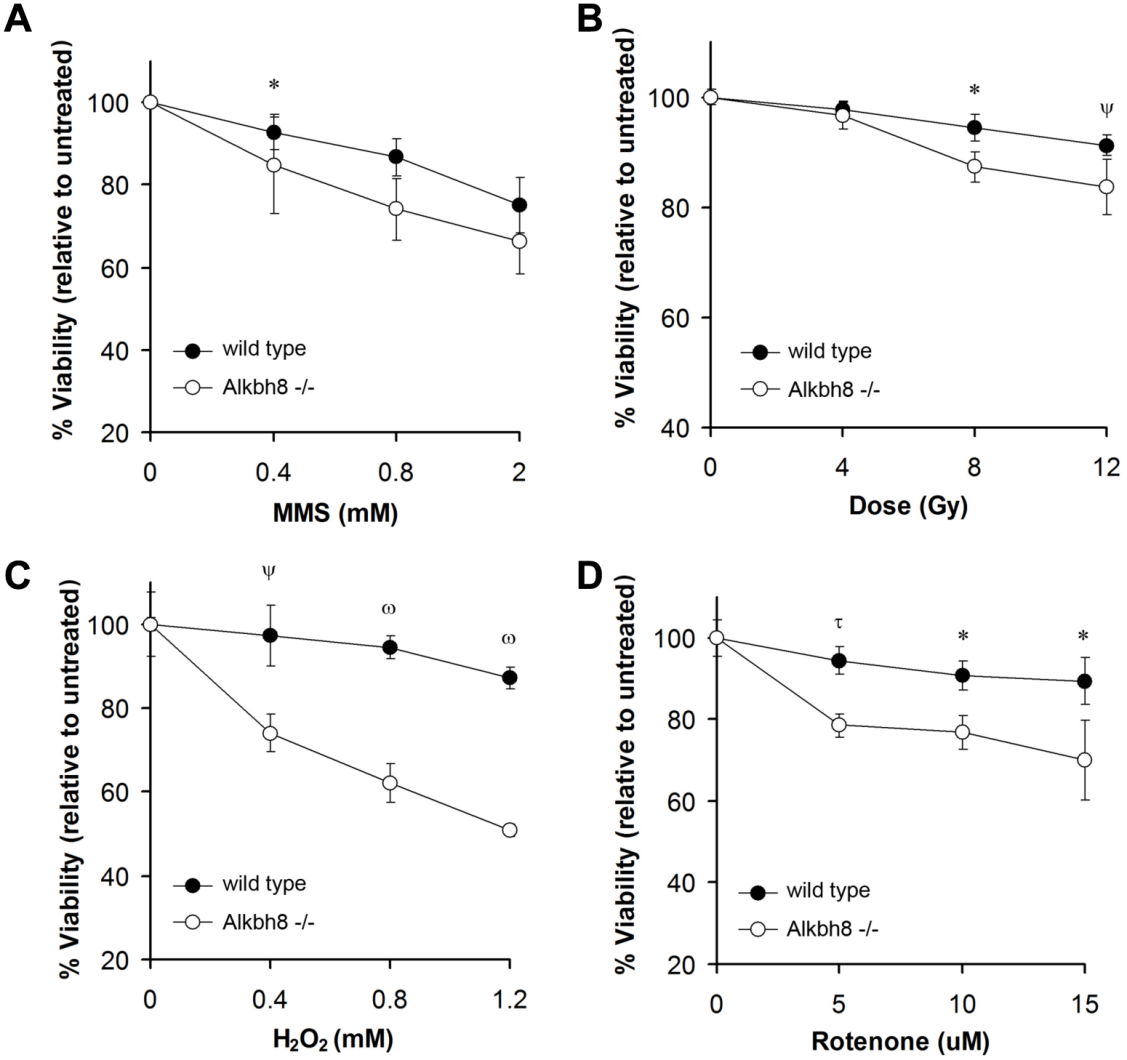
*Alkbh8* ^*-/-*^ MEFs are sensitive to DNA damaging agents. MEFs were either treated with **(A)** MMS, **(B)** exposed to γ-irradiation or treated with **(C)** H_2_O_2_ or **(D)** Rotenone and viability was assessed by enumerating PI or trypan blue negative cells 48 (MMS, irradiation, and Rotenone) or 72 (H_2_O_2_) h later. In all panels, error bars represent standard deviation (±STDV, n = 3) and significant differences in viability of wt and *Alkbh8* ^*-/-*^ MEFs were determined by Student’s t-test (^**τ**^p < 0.005, *p < 0.05, ^**ψ**^p < 0.01, ^ω^p < 0.001).

The increased strand breaks and activated DNA damage response in *Alkbh8* ^*-/-*^ MEFs suggested that Alkbh8 deficiency leads to increased levels of damaging agents and/or a compromised DNA damage response downstream of **γ**-H2AX phosphorylation. Often, defects in damage prevention or DNA damage-responses sensitize cells to DNA damaging agents. For example, ATM and BRCA1 mutant cell lines are highly sensitive to ionizing radiation [41, 42] and p53-deficient cells undergo greater DNA damage induced apoptosis in response to chemotherapeutic drugs that induce DNA strand breaks (*i*.*e*., topoisomerase inhibitors) in comparison to wild type p53 cells [43]. To help identify the type of damaging agent or the defective repair pathway in *Alkbh8* ^*-/-*^ MEFs we treated the cells with various DNA damaging agents. We observed an increased sensitivity of *Alkbh8* ^*-/-*^ MEFs, relative to wt MEFs, to MMS, ionizing irradiation, H_2_O_2_ and Rotenone (Fig 3). This sensitivity was particularly pronounced for the latter two compounds, which are both known to increase intracellular ROS, with Rotenone being an endogenous ROS inducer through its ability to perturb mitochondrial function. Taken together, these observations support a role for mammalian Alkbh8 in preventing ROS, oxidative DNA damage, or both, or participating in some aspect of the repair of oxidatively damaged DNA.

### Alkbh8 deficiency leads to oxidative stress under basal conditions

To gain further insight into Alkbh8 function, we also performed microarray analysis under basal conditions, with the idea that regulated transcripts could provide insight into the cellular role of Alkbh8. This analysis revealed that under normal growth conditions, there are significant differences in gene expression patterns between the *Alkbh8* ^*-/-*^ and wt MEFs: 88 transcripts were up-regulated and 7 transcripts were down-regulated >2-fold (p < 0.05) in *Alkbh8* ^*-/-*^ relative to wt MEFs (S1 Table, raw data will be deposited in ArrayExpress upon publication). Considering the sensitivity of *Alkbh8* ^*-/-*^ MEFs to DNA damaging agents that induce intracellular ROS, we were particularly intrigued to find that a subset of genes involved in the metabolic processing of ROS were up-regulated in *Alkbh8* ^*-/-*^ MEFs (Table 1), with notable examples being several glutathione S-transferases and superoxide dismutase 3, among others. We used a pathway-focused quantitative real-time polymerase chain reaction (qRT-PCR) arrays to validate many of these *Alkbh8* ^*-/-*^ up-regulated targets and to extend our observations to other transcripts known to operate in oxidative stress signaling. Consistent with our microarray analysis, we found that the transcripts of multiple genes that function in ROS detoxification are up-regulated in *Alkbh8* ^*-/-*^ MEFs, (S3 Fig) suggesting that these cells are subjected to higher levels of endogenous ROS. Notably, several glutathione S-transferases are up-regulated, which represent a class of genes with promoter antioxidant response elements (AREs) known to be Nrf2 transcription factor targets involved in maintaining cellular redox homeostasis [44].

**Table 1:** Microarray-based transcript analysis was performed on early passage (P3) *Alkbh8* ^*-/-*^ and wt MEFs. Fold change is the mean fluorescence intensity of *Alkbh8* ^*-/-*^ MEFs relative to wt MEFs (n = 3, p < 0.05).

Process	Fold change: KO/WT	Gene symbol	Description
Oxidative	2.15	Sqrdl	sulfide quinone reductase-like
	2.21	Adh7	alcohol dehydrogenase 7 (class IV)
	2.35	Sod3	superoxide dismutase 3
	2.38	Hspb2	heat shock protein 2
	2.77	Cbr2	carbonyl reductase 2
	3.17	Gsta3	glutathione S-transferase, alpha 3
	1.52	Gsto2	glutathione S-transferase omega 2
	1.55	Gstm7	glutathione S-transferase, mu 7
	1.61	Gstt1	glutathione S-transferase, theta 1
	2.19	Mgst1	microsomal glutathione S-transferase 1
Apoptosis	2.63	Perp	PERP, TP53 apoptosis effector
	2.03	Wisp2	WNT1 inducible signaling pathway protein 2

**Fig 4:**
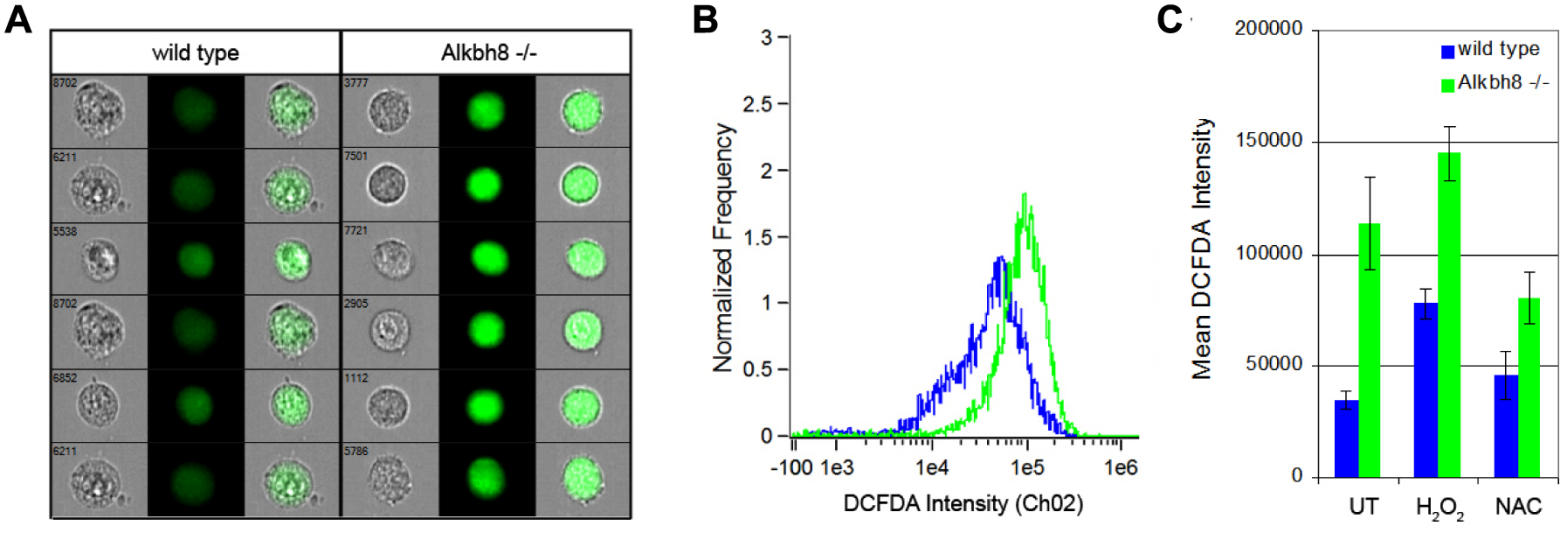
*Alkbh8* ^*-/-*^ cells have elevated reactive oxygen species (ROS). A) Intracellular ROS was measured in immortalized MEFs by DCFDA staining. Representative images are shown for cell batches taken from the peak frequency of DCFDA intensities for wt and *Alkbh8* ^*-/-*^ MEFs. B) The frequency of wt (blue) and *Alkbh8* ^*-/-*^ (green) cells exhibiting specific DCFDA intensities is plotted for a population of 6,000 cells. C) Intracellular ROS was measured in immortalized MEFs by DCFDA staining 6 h after the indicated treatments and is shown as median fluorescence intensity emitted by oxidized DCFDA at 517–527 nm +STDV, n = 3).

Our DNA damage, cell sensitivity and transcriptional analysis all support a role for Alkbh8 in the regulation of ROS levels or the repair of the resulting damage. To determine if the regulation of ROS levels is perturbed, we next measured overall levels of intracellular ROS in wt and *Alkbh8* ^*-/-*^ MEFs by staining cells with dichlorofluorescein diacetate (DCFDA) and used imaging flow cytometry to determine the median fluorescence intensity of our various cell populations. *Alkbh8* ^*-/-*^ MEFs had a higher median oxidized DCFDA fluorescence \intensity when compared to wt MEFs, indicating that these cells display increased intracellular ROS (Fig 4A and 4B). This effect was exacerbated after treatment of the cells with H_2_O_2_. Notably the increased ROS observed in *Alkbh8* ^*-/-*^ MEFs could be rescued by antioxidant treatment with N-acetylcysteine (NAC) (Fig 4C). The increased ROS phenotype was also apparent in immortalized *Alkbh8* ^*-/-*^ cells, which were established concurrent with primary MEF experiments (results not shown). Our DCFDA results support the idea that Alkbh8 plays an important role in the detoxification of ROS.

### Compromised ROS response and decreased selenocysteine protein expression in *Alkbh8* ^*-/-*^ MEFs

**Fig 5:**
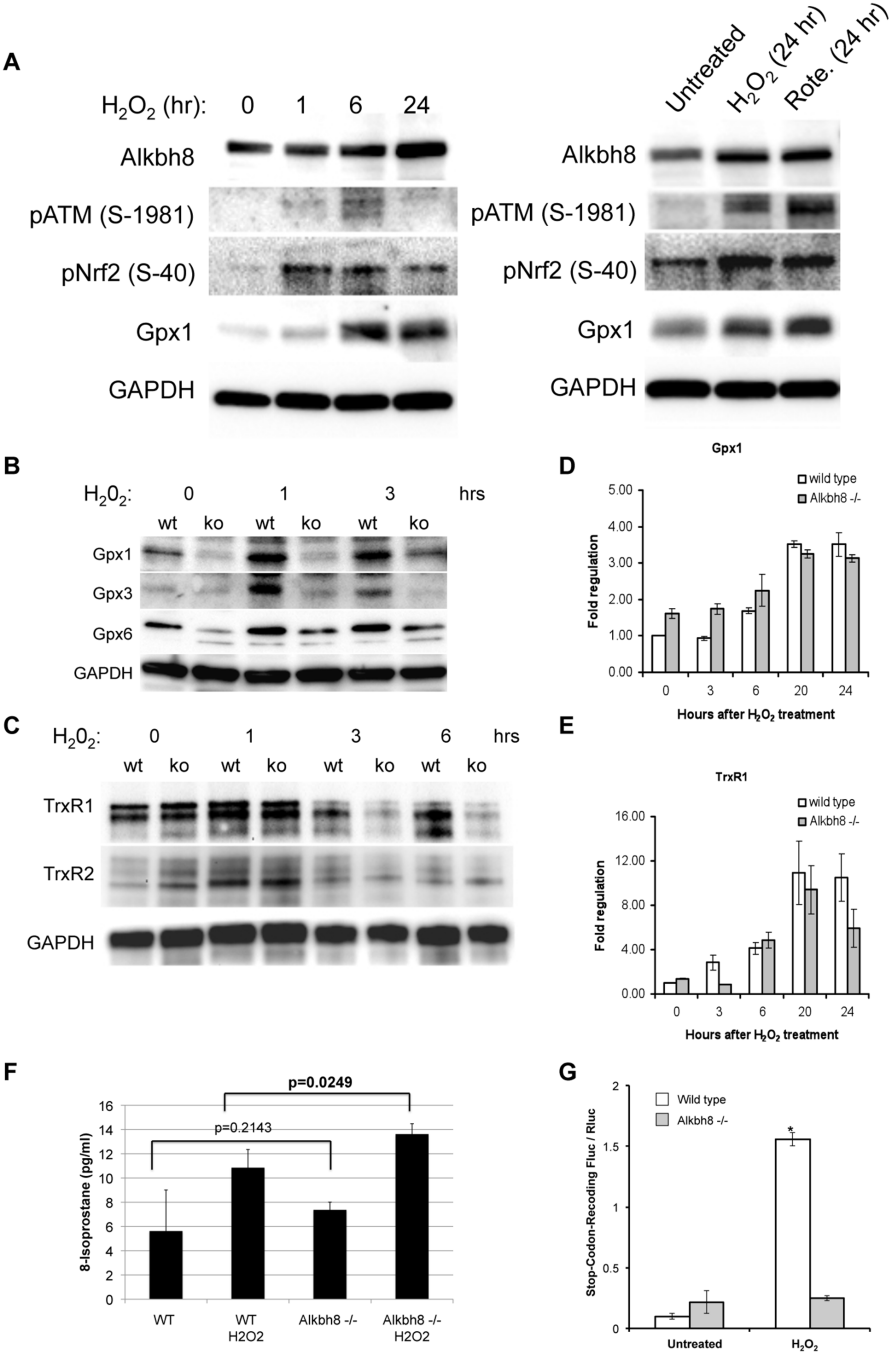
Compromised ROS response and stop-codon recoding with decreased Gpx and TrxR1 protein levels and activity in *Alkbh8* ^*-/-*^ MEFs. Wt and *Alkbh8* ^*-/-*^ MEFs were treated as indicated and then prepared for western blot analysis with **A)** anti-Alkbh8, anti-Nrf2, anti-ATM S15 phosphorylated **B)** anti-Gpx, **C)** and anti-TrxR antibodies. Anti-TrXR1 antibody (Peirce PAS-28886) detects TrxR1 at 67 kD and a cross-reacting (higher MW) band in mouse cells. For **A—C**, anti-GAPDH antibodies were used to as loading controls. **D)** qRT-PCR analysis of Gpx1 transcript levels. **E)** qRT-PCR analysis of TrxR1 transcript levels. **F)** Lipid peroxidation levels were measured in whole cell lysates prepared from wt and *Alkbh8* ^*-/-*^ MEFs under basal and H_2_O_2_-treated conditions. **G)** Stop-codon recoding was measured using the DualLuc-Gpx1 reporter system in wt and *Alkbh8* ^*-/-*^ MEFs under basal and H_2_O_2_-treated conditions. Statistical significance (p < 0.05) of biological replicates (N = 3) was measured using the Student’s t-Test.

We used immunoblot approaches to determine if Alkbh8 gene expression is regulated in response to H_2_O_2_ exposure. Using wild type MEFs (both immortalized and primary) we demonstrate that Alkbh8 protein levels are induced by H_2_O_2_, further supporting a role for this enzyme in oxidative-induced stress response (Fig 5A). As Alkbh8-catalyzed tRNA modifications play a role in stop-codon reprogramming for efficient Gpx1 translation [15, 27, 29] we next analyzed protein expression levels for multiple selenocysteine containing glutathione peroxidase proteins (Gpx1, Gpx3 and Gpx6) in wild type and *Alkbh8* ^*-/-*^ MEFs. Notably, the protein expression of several Gpx family members was affected by Alkbh8 status (Fig 5B). Specifically, the protein levels of Gpx1 and Gpx6 were decreased in *Alkbh8* ^*-/-*^ cells under basal growth conditions; Gpx1, Gpx3 and 6 expression were all induced by oxidative-stress (*i*.*e*., H_2_O_2_), and this induction was markedly attenuated in *Alkbh8* ^*-/-*^ MEFs, suggesting that Gpx3 in MEFs is also acting in antioxidant defense in addition to playing a housekeeping role. Moreover, the decreased Gpx protein levels correlated with a decrease in Gpx activity in *Alkbh8* ^*-/-*^ MEFs relative to wt MEFs (S4 Fig). The cytosolic TrxR1 and mitochondrial TrxR2 proteins levels were also analyzed in wt and *Alkbh8* ^*-/-*^ MEFs under basal and H_2_O_2_ induction conditions. While the levels of both TrxR1 and TrxR2 were similar in wt and *Alkbh8* ^*-/-*^ MEFs under untreated conditions, we did observe a modest decrease in TrxR1 in *Alkbh8* ^*-/-*^ MEFs three and six hours after H_2_O_2_ treatment (Fig 5C). We did not observe a noticeable decrease in TrxR2 in *Alkbh8* ^*-/-*^ MEFs under any condition, relative to wt, thus we can only link Alkbh8 to the regulation of TrxR1 in the cytosol. To further link Alkbh8 to the regulation of Gpx1 and TrxR1 we introduced a control plasmid (pcDNA3.1-EV) and an Alkbh8 expression plasmid (pcDNA3.1-Alkbh8) into our *Alkbh8* ^*-/-*^ MEFs and analyzed for protein levels under basal conditions. We determined that re-expression of Alkbh8 in the *Alkbh8* ^*-/-*^ MEFs rescued Gpx1 and TrxR1 protein levels, while having little effect on TrxR2 levels (S5 Fig). qRT-PCR analysis of Gpx1 and TrxR1 transcripts in our MEF models demonstrates little difference in mRNA levels between wt and *Alkbh8* ^*-/-*^ (Fig 5D and 5E). Together the data support the idea that the observed decreases in selenocysteine protein expression in *Alkbh8* ^*-/-*^ MEFs is due an Alkbh8 deficiency that leads to a defect in post-transcriptional regulation of gene expression.

The decreased selenoprotein levels observed in *Alkbh8* ^*-/-*^ MEFs would suggest decreased Gpx4 levels and a subsequent increase in lipid peroxidation products. As such, we identified a significant increase (p < 0.03) in 8-isoprostane levels in *Alkbh8* ^*-/-*^ MEFs, after H_2_O_2_ exposure (Fig 5F). Most likely the increased lipid peroxidation is due to deficiency in Gpx4 levels, which detoxifies these ROS products. We were unable to detect any Gpx4 in wt MEFs by immunoblots (data not shown). Our results support that Alkbh8 is required for optimal protein expression of a number of selenocysteine proteins (Gpx1, Gpx3, Gpx6 and TrxR1) and possibly Gpx4.

The transcriptional and protein level data specific to selenoproteins supports the idea that stop codon recoding and selenocysteine incorporation is corrupted in *Alkbh8* ^*-/-*^ MEFs, relative to wt. We used a reporter system to further support the idea that Alkbh8 is a key node that regulates the response to ROS. Stop codon recoding was measured in wt and *Alkbh8* ^*-/-*^ MEFs using the DualLuc-Gpx1 reporter system developed by Falnes and colleagues [15]. The dual luciferase construct, pCMV-Script_DualLuc-GPx-SECIS, encodes tandem *Renilla reniformis* and firefly (*Photinus pyralis*) luciferase enzymes separated by a single UGA codon. We observed little difference in reporter activity when comparing the wt and *Alkbh8* ^*-/-*^ MEFs under untreated conditions (Fig 5G). In response to H_2_O_2_ treatment we observed a ~12-fold increase in reporter activity in wt MEFs, supporting the idea that stop codon recoding is increased in response to stress. In contrast we observed little H_2_O_2_ induced increase in reporter activity in *Alkbh8* ^*-/-*^ MEFs, which represents a significant (p < 0.05) ~6-fold decrease in reporter activity relative to wt MEFs under H_2_O_2_ conditions (Fig 5G). Together our immunoblot, qPCR and reporter data support the idea that stress induced translation of selenoproteins is disrupted in the *Alkbh8* ^*-/-*^ MEFs.

### The H_2_O_2_ induced increase in mcm^5^Um is corrupted in Alkbh8^-/-^ MEFs

**Fig 6:**
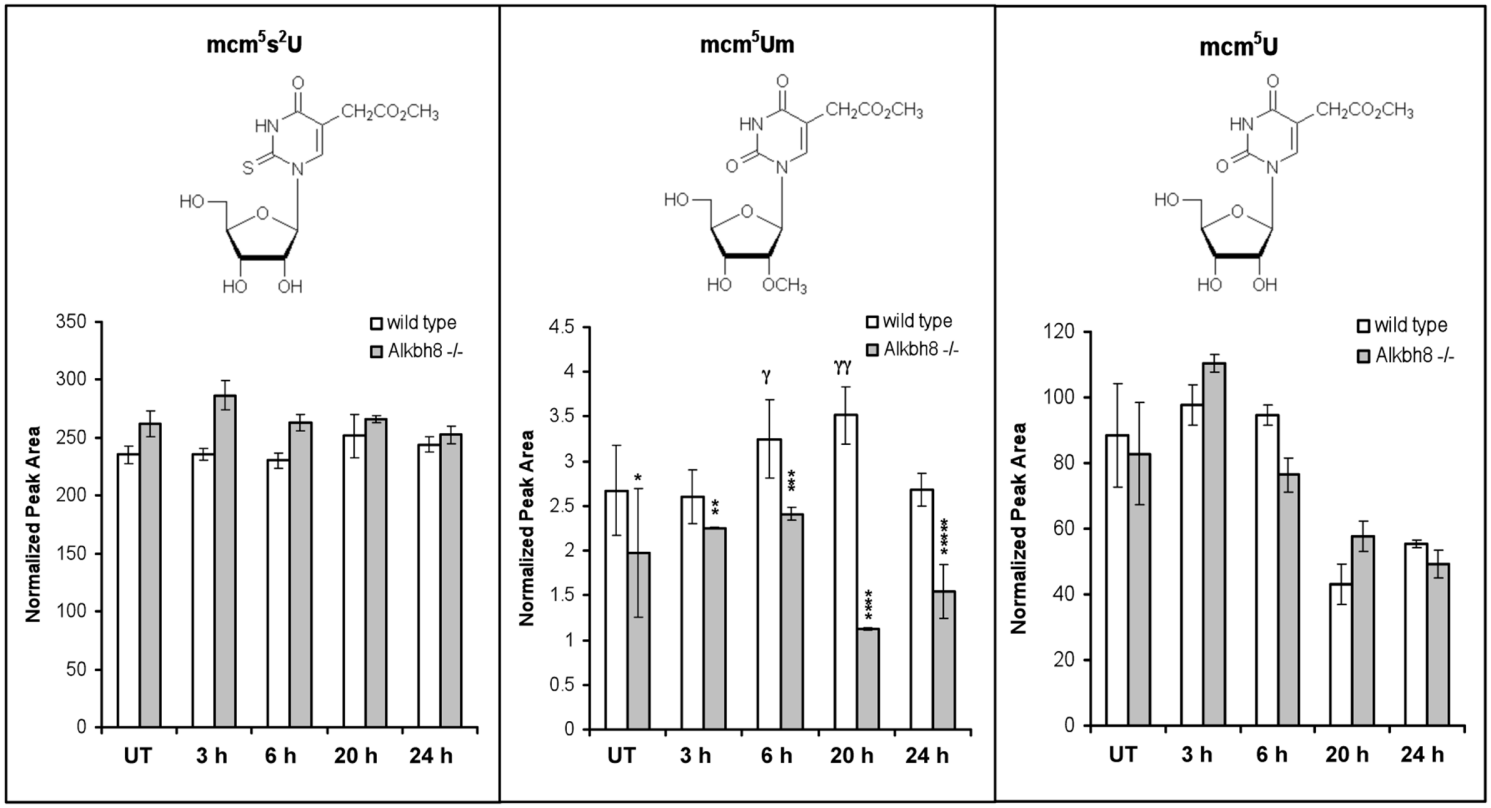
Ribonucleoside modifications in wild type and *Alkbh8* ^*-/-*^ mice after oxidative stress. Wt and *Alkbh8* ^*-/-*^ MEFs were either left untreated (UT) or exposed to 1.2 mM of H_2_O_2_ for 1 hour. The levels of mcm^5^s^2^U, mcm^5^Um and mcm^5^U ribonucleoside modifications were identified by HPLC-coupled mass spectrometry and quantified by integrating the normalized peak area intensity for each signal at the indicated post-exposure time points. Significant differences in mcm^5^Um modifications were determined by the Student’s t-test: UT, *Alkbh8* ^*-/-*^ (n = 6) versus wt (n = 5), *p < 0.05; 3 h, *Alkbh8* ^*-/-*^ (n = 3) versus wt (n = 3), **p < 0.02; 6 h, *Alkbh8* ^*-/-*^ (n = 3) versus wt (n = 3), ***p < 0.002; 20 h, *Alkbh8* ^*-/-*^ (n = 2) versus wt (n = 2), ****p < 0.01; 24 h, *Alkbh8* ^-/-^ (n = 3) versus wt (n = 2), *****p < 0.001. UT, 6 h, wt (n = 3) versus 0 hours, wt ^**γ**^p < 0.02; 20 hours, wt (n = 2) versus 0 h, wt (n = 5) ^**γγ**^p < 0.01.

The observed H_2_O_2_-induced increases in Alkbh8, Gpx1, Gpx3, and Gpx6 proteins in wt MEFs suggest that the associated tRNA modifications required for the translation of selenocysteine containing proteins are also increased in response to elevated ROS levels. Further the decreased Gpx1, Gpx3 and Gpx6 in in *Alkbh8* ^*-/-*^ MEFs suggests that tRNA modification is affected, relative to wt. To investigate these possibilities, we used our established LC-MS/MS approach [14, 17, 19, 36] with mass transitions specified in Table 2 to quantitate the basal and H_2_O_2_-induced levels of mcm^5^U, mcm^5^s^2^U and mcm^5^Um in wt and *Alkbh8* ^*-/-*^ MEFs (Fig 6). When comparing wt and *Alkbh8* ^*-/-*^ MEFs, we observed little difference in mcm^5^U and mcm^5^s^2^U levels under both basal and H_2_O_2_ treated conditions. In contrast, we observed a significant (p < 0.05) difference in mcm^5^Um in wt and *Alkbh8* ^*-/-*^ MEFs under basal conditions and after H_2_O_2_ treatment. We have also quantitated mcm^5^-base modifications in the livers of wt and *Alkbh8* ^*-/-*^ animals and observed analogous results to our MEF’s. Specifically we observed similar levels of the mcm^5^U and mcm^5^s^2^U modifications in wt and *Alkbh8* ^*-/-*^ livers and significantly decreased (p < 0.05) levels of the mcm^5^Um modification in the Alkbh8^-/-^ vs. wt livers (S6 Fig). After exposure to ROS, the most obvious difference in mcm^5^Um levels in MEFs was seen 20 h after H_2_O_2_ treatment, when the wt cells had ~3.5-fold higher levels of this wobble base modification only found on tRNA^UGA-SEC^, relative to *Alkbh8* ^*-/-*^ MEFs. The 20-hour post-H_2_O_2_ time point also represents the peak levels of mcm^5^Um for wt MEFs, and lowest levels for *Alkbh8* ^*-/-*^ MEFs, further demonstrating a significant Alkbh8-dependent increase.

**Table 2:** Settings of the Agilent 6430 triple quadrupole mass spectrometer for ribonucleoside analysis.

Molecule	*m/z* at Q1	*m/z* at Q3	Fragmentor Voltage (V)	Collision Energy (eV)	Dwell time (ms)	Retention Time (min)
mcm^5^S^2^U	333	201	80	4	200	11.7
	333	169	100	12	200	11.7
mcm^5^Um	331	185	84	4	200	11.3
mcm^5^U	317	185	94	4	200	7.8
	317	153	94	16	200	7.8
[^15^N]_5_dA	257	141	90	10	200	5.1

## Discussion

### Alkbh8 is a key node in the ROS response network

**Fig 7:**
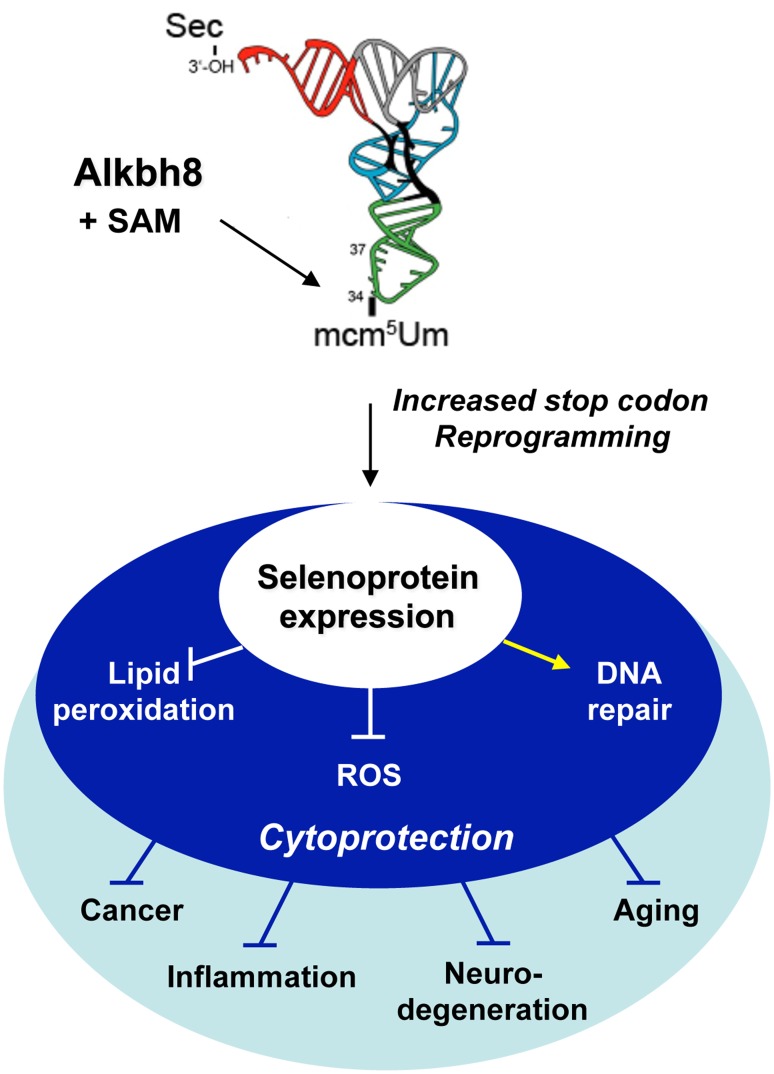
Model for Alkbh8 as a translational regulator of an ROS response node. Alkbh8-directed cytoprotective responses through stop codon reprogramming for selenoprotein expression, and implications for disease prevention.

We are the first group to demonstrate that *Alkbh8* ^*-/-*^ MEFs have elevated intracellular reactive oxygen species (ROS) and increased DNA damage levels, with these phenotypes linked to reduced Gpx1, Gpx3, Gpx6 and TrxR1 protein levels. Further we report that lipid peroxidation products are higher in *Alkbh8* ^*-/-*^ MEFs after H_2_O_2_ treatment, relative to wt, supporting a regulatory link between Alkbh8 and Gpx4. Importantly we demonstrate that there is an increase in Alkbh8 protein and mcm^5^Um levels in response to H_2_O_2_ and identify a key regulatory pathway in the response to ROS. The ROS stress phenotype observed in our *Alkbh8* ^*-/-*^ MEFs is due to defective stop codon reprogramming, leading to sub-optimal selenocysteine protein expression. Alkbh8-regulated ROS and lipid detoxification systems provide cytoprotection against ROS-stress (Fig 7). Faced with increased ROS levels, it makes sense for the cell to increase Alkbh8 and mcm^5^Um levels to increase the amount of Gpx1, Gpx3, Gpx4, Gpx6 and TrxR1, so as to increase H_2_O_2_ and lipid peroxidation detoxification capacity. Reducing the levels of H_2_O_2_ and other ROS will clearly prevent DNA and protein damage and reduce cell death. Alkbh8 can thus be thought of as a regulator of a damage mitigation system that protects DNA from ROS offenders. Further, the removal of lipid peroxidation products should prevent DNA mutation, since lipid peroxidation products like malondialdehyde have been demonstrated to cause DNA damage that promotes insertion, deletion and basepair mutations in human cells [45]. Thus, we have established a cytoprotective role for Alkbh8. Based on the increased DNA damage, lipid peroxidation and the mcm^5^Um-dependent regulation of Gpx1, Gpx3 and Gpx6, there are suggested anti-mutagenic roles for Alkbh8 via the prevention of DNA damage such as 8-oxoguanine and M_1_G (pyrimidopurin-10(3H)-one). Our suggestion is supported by the documented increase in mutagenesis that is observed in catalase deficient bacterial cells [46].

*In vivo* mouse studies have previously demonstrated that Alkbh8 deletion results in a lack of mcm^5^U, mcm^5^s^2^U and mcm^5^Um ribonucleotide modifications, together with a concomitant decrease in basal Gpx1 [15]. Human *ALKBH8* knock down models demonstrate decreased mcm^5^U levels, further supporting a role for mammalian Alkbh8 in translational decoding [14]. Our model and study is unique in that the *Alkbh8* ^*-/-*^ MEFs generated by insertional mutagenesis only show a decrease in mcm^5^Um levels. Surprisingly, we did not see a decrease in mcm^5^U or mcm^5^s^2^U levels in our *Alkbh8* ^*-/-*^ MEFs and livers. This is in contrast to the data reported in the liver, brain and testis for the Alkbh8 knockout model created by a conventional gene targeting strategy that removed exons 9–10 and thus part of the protein. We also did not observe a decrease in mcm^5^U that was reported in human embryonic kidney cells that are knocked down for ALKBH8 [14, 15]. The difference between our model and the previous reports could be due to the design of our gene trap construct, which is an insertion of a ß-galactosidase/neomycin resistance (ßgeo) insertion cassette that also provides an in frame stop codon. Despite a 30-fold decrease in *Alkbh8* transcript levels in our gene-trapped MEFs, a low level of Alkbh8 transcript expression (*i*.*e*., via stop-codon read through) (S1C Fig) could produce an amount of fusion protein sufficient for catalyzing the mcm^5^U and mcm^5^s^2^U modifications. There is clear evidence that the level of Alkbh8 or activity in the *Alkbh8* ^*-/-*^ MEFs and livers is insufficient for the production of the mcm^5^Um modification in response to oxidative stress (Fig 7). This highlights the possibility of Alkbh8 being the methyltransferase responsible for ROS induced ribose methylation, but detailed biochemical experiments are needed to confirm this possibility. Together, these results point to a role for Alkbh8 and mcm^5^Um modification in oxidative-stress induced translation of Gpx1, Gpx3, Gpx4, Gpx6 and TrxR1, and is consistent with the idea that the mcm^5^Um modification is particularly important for the optimal expression of stress-related selenocysteine proteins [15, 24–28].

TrxR1 is another selenocysteine-containing protein that requires Alkbh8 for optimal expression in response to H_2_O_2_ exposure. TrxR1 can be directly linked to ROS mitigation and the prevention of DNA damage. TrxR1 has Trx as a substrate, and reduced Trx can then catalyze disulfide bond reduction in a number of substrate proteins, including those that function as anti-oxidants and ribonucleotide reductase. The rate-limiting step of deoxyribonucleotide synthesis is catalyzed by ribonucleotide reductase enzymes with the associated dNTPs being required for efficient DNA repair and replication [47]. Many types of cancer cells have been shown to up-regulate the expression of Trx, which could optimize ribonucleotide reductase activity to supply the large concentration of dNTPs required for a rapidly dividing cell population [47–50]. Notably, the yeast Alkbh8 homolog Trm9 regulates the expression of the large ribonucleotide reductase proteins (Rnr1 and Rnr3) by enhancing the decoding of specific codons found in the corresponding transcripts [18, 20]. It is intriguing to speculate that mammalian Alkbh8 arrives at the same molecular endpoint (*i*.*e*., Alkbh8-TrxR1-Trx connection to enhance RNR activity) albeit by a different mcm^5^-dependent mechanism (*i*.*e*., stop codon recoding for TrxR1). A further connection between Alkbh8 and the DNA damage response comes from the observation that human *ALKBH8* transcript expression is induced by various forms of DNA damage and that this induction is attenuated in ataxia telangiectasia (ATM)-deficient cells [14].

The biological pathways that regulate the gene expression of other Alkbh family members are linked to cell stress responses. For example, Alkbh5 has recently been shown to be a direct target of the transcription factor, hypoxia inducible factor 1 (HIF1), suggesting a role for 2OG-Fe(II) oxygenases in hypoxic stress responses [51]. Most recently, Fu et al. (2013), provided evidence that Alkbh7 is a mitochondrial protein essential for a form of alkylation DNA damage-induced programmed necrosis, involving mitochondrial destabilization, ROS generation and complete cellular energy depletion [52]. Using budding yeast, it has been demonstrated that H_2_O_2_ will regulate the levels of many tRNA modifications [19]. Our findings that H_2_O_2_ induces Alkbh8 expression and increases mcm^5^Um levels in wild-type MEFs demonstrate that tRNA modification levels increase in mammalian cells in response to ROS stress. ROS-induced reprogramming of tRNA modifications has now been shown in both mammalian and yeast cells, suggesting that increases in tRNA modification in response to stress will be a common theme found across phylogeny. The *Alkbh8* ^*-/-*^ time course data for mcm^5^Um suggests that this RNA modification is actively depleted in response to H_2_O_2_ treatment (Fig 6). The decrease in mcm^5^Um in *Alkbh8* ^*-/-*^ MEFS after H_2_O_2_ exposure could prevent or decrease the translation of some stress-response selenoproteins and could be due to the degradation of the associated tRNA or some active processing of the modification to another chemical form.

### Alkbh8 deregulation promotes phenotypes linked to disease

All of the mis-regulated Gpx and TrxR proteins in *Alkbh8* ^*-/-*^ MEFs are selenoproteins. There are strong links between selenocysteine-mediated ROS detoxification processes and disease. Dietary selenium has been linked to cancer prevention, proper immune function, anti-aging and other pathophysiological processes [53–56]. Further, Gpx deficiency leads to cancer predisposition [57] and *in vivo* sensitivity to oxidant-inducing agents is well documented for mice lacking selenocysteine-containing proteins such as Gpx1 and Gpx4 [58–60]. Our results in MEFs predict that *Alkbh8* ^*-/-*^ mice will have an increased sensitivity to ROS inducing agents and may be predisposed to develop diseases linked to aberrant ROS production (*i*.*e*., neurodegenerative and inflammatory diseases or cancer) (Fig 7). Therefore, significant future studies will involve aging and exposure studies of *Alkbh8* ^*-/-*^ mice to determine tumor rates relative to wild-type mice.

In conclusion, we have defined a mechanistic role for Alkbh8 in the oxidative stress response, which has implications for cancer development and other pathologies arising from defective anti-oxidant and redox cycling defenses. Altered Alkbh8 expression has been implicated in cancer etiology, as abnormally high Alkbh8 expression is associated with an aggressive cancer phenotype, tumor angiogenesis and metastasis [61]. Although Alkbh8 acts cytoprotectively as part of an anti-oxidant defense system in untransformed cells, augmented Alkbh8 expression to tolerate increased ROS levels associated with some cell transformations could represent an adaptive response. Cancer cells are known to thrive under inherently high oxidative stress conditions relative to normal cells, and the cancer stem cells that arise from these are known to have enhanced ROS defenses [62–64]. Interestingly, cancer cell adaptation to high ROS has been observed for other anti-oxidant systems, like the superoxide dismutases [64]. In the broader scheme of cellular signaling, Alkbh8 represents a new link between translation-based regulatory mechanisms (*i*.*e*., stop codon reprogramming) and stress responses, with likely implications in cancer.

## Supporting Information

S1 Table Transcripts Differentially Regulated in *Alkbh8* ^*-/-*^ MEFs Relative to WT. Affymetrix gene chips (N = 3) were used to quantitate transcript levels in wt and *Alkbh8* ^*-/-*^ MEFs. 205 transcripts that were significantly (p < 0.05) increased or decreased 1.5-fold in *Alkbh8* ^*-/-*^ MEFs, relative to wt, are shown.
(PDF)

S1 Fig Schematic of the *Alkbh8* ^*-/-*^ allele. **A)** Embryonic stem cells with a gene trapped Alkbh8 allele, designated Alkbh8^Gt(RRY122)Byg^, were obtained from BayGenomics and used for injection into C57BL/6 blastocysts for the creation of Alkbh8-deficient heterozygous mouse [34]. Specific ß-galactosidase/neomycin resistance (ßgeo) vector insertion was mapped to chromosome 9:3335231–3385847 and occurs within intron 7 of the mouse Alkbh8 gene. **B)** PCR amplification with ßgeo specific primer sequences confirmed the presence of the ßgeo cassette in the ES cells. **(C)** Alkbh8 expression in MEFs was analyzed by qRT-PCR to establish gene copy number **(D)** and western blotting to confirm Alkbh8 protein expression in wt but not *Alkbh8* ^*-/-*^ MEFs that were immortalized by sequential passage. Human embryonic kidney cells that over-expressed a full-length murine Alkbh8 expression plasmid (HEK + Alkbh8) served as a positive control.
(PDF)

S2 Fig Cell cycle analysis of wild type and *Alkbh8* ^*-/-*^ MEFs. MEFs were stained with propidium iodide and the percentage of cells in G1/G0, S or G2/M phases of the cell cycle was determined by flow cytometry analysis of DNA content (±STDV, n = 3).
(PDF)

S3 Fig qRT-PCR confirmation of transcripts regulated in *Alkbh8* ^*-/-*^ MEFs. Microarray gene targets were confirmed as up-regulated targets by qRT-PCR gene expression analysis using RT2 Profiler (Qiagen) arrays of gene targets involved in the oxidative stress response.
(PDF)

S4 Fig *Alkbh8* ^*-/-*^ MEFs have decreased Gpx activity. Gpx activity was measured in whole cell lysates prepared from wt and *Alkbh8* ^*-/-*^ MEFs under basal growth conditions.
(PDF)

S5 Fig Rescue of Gpx1 and TrxR1 protein levels in *Alkbh8* ^*-/-*^ MEFs re-expressing Alkbh8. *Alkbh8* ^*-/-*^ MEFs were necleofected with empty vector or an Alkbh8 expression vector. Immunoblot analysis was performed as described in Fig 5.
(PDF)

S6 Fig *Alkbh8* ^*-/-*^ livers have decreased mcm^5^Um and Gpx protein levels. Protein and tRNA fractions were isolated from the livers of littermate-matched wild type and *Alkbh8* ^*-/-*^ mice. **(A)** Mcm^5^-uridine based modifications were measured (N = 4) from the livers of wt and *Alkbh8* ^*-/-*^ mice by HPLC-coupled mass spectrometry and quantified by integrating the normalized peak area intensity for each signal at the indicated post-exposure time points. Significant differences in mcm^5^Um modifications was determined by the Student’s t-test. **(B)** Gpx and Gapdh protein levels were analyzed in mouse liver extracts by immunoblots.
(PDF)
