# Supplementary material for: Alkbh8 Regulates Selenocysteine-Protein Expression to Protect against Reactive Oxygen Species Damage
Source: PLoS One. 2015 Jul 6;10(7):e0131335. doi: 10.1371/journal.pone.0131335 (PMC4492958; doi:10.1371/journal.pone.0131335)
Supplement: S1 Table — Affymetrix gene chips (N = 3) were used to quantitate transcript levels in wt and Alkbh8 -/- MEFs. 205 transcripts that were significantly (p < 0.05) increased or decreased 1.5-fold in Alkbh8 -/- MEFs, relative to wt, are shown. (PDF) [file pone.0131335.s001.pdf]

Table S1. Transcripts Differentially Regulated in Alkbh8 $^{-/-}$ MEFs Relative to WT

Transcripts Cluster Id	[KO](normalized)	[WT](normalized)	p-value	regulation	FC KO/WT	genesymbol	genbank	gene description
10496359	7.90	9.42	0.01234	down	-2.88	Emcn	BC003706	endomucin
10498441	7.43	8.89	0.00776	down	-2.75	Gpr149	BC119599	G protein-coupled receptor 149
10576581	7.89	9.30	0.00947	down	-2.66	Kcnk1	AF033017	potassium channel, subfamily K, member 1
10399820	9.07	10.39	0.0126	down	-2.49	Acp1	BC112413	acid phosphatase 1, soluble | similar to acid phosphatase 1, soluble
10437055	7.28	8.40	0.00339	down	-2.17	Sim2	D64135	single-minded homolog 2 (Drosophila)
10533246	7.53	8.63	0.00316	down	-2.15	Oas1a	BC043339|BC013715	2'-5' oligoadenylate synthetase 1G | 2'-5' oligoadenylate synthetase 1A
10603573	7.08	8.09	0.00656	down	-2.01	Sytl5	AB098160	synaptotagmin-like 5
10438791	8.30	9.29	0.00217	down	-1.99	Uts2d	AY321315	urotensin 2 domain containing
10582925	8.32	9.26	6.31E-04	down	-1.92	Alkbh8	BC050863	alkB, alkylation repair homolog 8 (E. coli)
10500938	7.77	8.70	0.00739	down	-1.91	Wnt2b	BC119276	wingless related MMTV integration site 2b
10462390	7.16	8.03	0.00324	down	-1.83	Cd274	BC066841	CD274 antigen
10399725	10.86	11.65	8.90E-04	down	-1.73	Sox11	AF009414	SRY-box containing gene 11
10399586	9.71	10.48	0.00168	down	-1.71	ZT2	AJ005350	zinc finger protein 125
10427303	9.64	10.40	0.0037	down	-1.70	Hoxc4	X69019	homeo box C4
10411226	10.01	10.72	0.01535	down	-1.64	F2rl1	BC025432	coagulation factor II (thrombin) receptor-like 1
10380622	10.37	11.06	0.01543	down	-1.62	Hoxb9	BC100743	homeo box B9
10502451	8.47	9.16	0.00334	down	-1.61	Bmpr1b	BC065143	bone morphogenetic protein receptor, type 1B
10414805	7.51	8.19	0.01512	down	-1.61	Tcra|Tcr alpha|Tcralpha	AK131826	T cell receptor alpha variable 13D-4
10356475	9.76	10.45	0.00527	down	-1.61	Arl4c	BC055769	ADP-ribosylation factor-like 4C
10431915	10.10	10.77	0.0021	down	-1.58	Slc38a4	AY027919	solute carrier family 38, member 4
10461152	10.34	10.98	0.00359	down	-1.56	Snhg1	AK051045	small nucleolar RNA host gene (non-protein coding) 1
10435075	10.18	10.81	1.39E-04	down	-1.55	Tfrc	BC054522	transferrin receptor
10427290	9.22	9.82	0.00304	down	-1.52	Hoxc8	X07646	homeo box C8
10384985	9.63	9.04	0.00848	up	1.50	Rhbdf1	BC027346	rhomboid family 1 (Drosophila)
10358339	7.80	7.21	1.84E-04	up	1.50	Cfh	M12660	complement component factor h
10405343	9.44	8.84	0.0087	up	1.51	Tspan17	BC010346	tetraspanin 17
10598833	7.52	6.92	6.95E-04	up	1.52	Chst7	BC019204	carbohydrate (N-acetylglucosamino) sulfotransferase 7
10464425	8.74	8.13	0.0062	up	1.52	Grk5	AF040746	G protein-coupled receptor kinase 5
10463844	9.30	8.69	0.01589	up	1.52	Gsto2	BC030371	glutathione S-transferase omega 2
10498576	12.52	11.91	8.09E-04	up	1.53	Lxn	D88769	latexin
10381445	8.88	8.27	0.00401	up	1.53	Tmem106a	BC022145	transmembrane protein 106A
10399005	11.08	10.47	0.00643	up	1.53	Crip1	BC031922	cysteine-rich protein 1 (intestinal)
10541496	11.78	11.16	0.01351	up	1.53	Mfap5	AF180805	microfibrillar associated protein 5
10595979	11.29	10.68	0.00833	up	1.53	Mras	AK050469	muscle and microspikes RAS
10526459	8.64	8.01	0.00401	up	1.54	Rasa4	BC049381	RAS p21 protein activator 4
10373740	9.10	8.48	0.00805	up	1.54	Pik3ip1	AY522649	phosphoinositide-3-kinase interacting protein 1
10501199	8.86	8.23	0.0029	up	1.55	Gstm7	BC051924	glutathione S-transferase, mu 7
10414360	12.17	11.53	0.01022	up	1.55	Lgals3	BC132328	lectin, galactose binding, soluble 3
10432918	8.34	7.71	0.00563	up	1.55	Krt8	M21836	keratin 8
10372781	8.16	7.53	0.00582	up	1.55	Irak3	BC120829	interleukin-1 receptor-associated kinase 3
10513208	10.35	9.72	0.01476	up	1.55	Svep1	AF206329	sushi, von Willebrand factor type A, EGF and pentraxin domain containing 1
10475437	8.40	7.75	0.01456	up	1.56	Sord	BC092291	sorbitol dehydrogenase
10546891	9.55	8.91	0.00801	up	1.57	Camk1	BC014825	calcium/calmodulin-dependent protein kinase I
10580537	8.80	8.14	0.01422	up	1.58	Aktip	BC008130	thymoma viral proto-oncogene 1 interacting protein
10537227	8.95	8.28	9.99E-04	up	1.58	Tmem140	BC023802	transmembrane protein 140
10470529	9.13	8.47	0.00611	up	1.58	Olfm1	D78262	olfactomedin 1
10599654	9.62	8.96	0.01478	up	1.59	Cxx1c	BC031701	CAAX box 1 homolog C (human)
10454254	8.87	8.20	2.39E-05	up	1.60	Dtna	BC040364	dystrobrevin alpha
10346374	7.94	7.26	0.01594	up	1.60	Aox1	AB017482	aldehyde oxidase 1
10371607	8.89	8.21	0.00906	up	1.60	Dram1	BC021433	RIKEN cDNA 1200002N14 gene
10504417	8.79	8.11	0.01101	up	1.60	Hrct1	AK014425	RIKEN cDNA 3830408D24 gene
10584653	8.17	7.49	0.00122	up	1.60	CTRP5|Mfrp	AF469650	C1q and tumor necrosis factor related protein 5 | membrane-type frizzled-related protein
10554819	7.32	6.63	0.01381	up	1.61	Me3	BC099935	malic enzyme 3, NADP(+)-dependent, mitochondrial
10457071	11.26	10.57	0.0011	up	1.61	Cyb5	BC024341	cytochrome b-5
10370000	7.58	6.89	0.01066	up	1.61	Gstt1	BC012254	glutathione S-transferase, theta 1
10470959	9.48	8.79	0.01178	up	1.62	Phyhd1|Lrrc8a	BC039982|BC048152	phytanoyl-CoA dioxygenase domain containing 1 | leucine rich repeat containing 8A
10538802	7.22	6.53	2.18E-04	up	1.62	A930038C07Rik	BC047154	RIKEN cDNA A930038C07 gene
10420631	10.42	9.73	0.00102	up	1.62	Ebpl	BC027422	emopamil binding protein-like
10407955	9.25	8.55	0.00968	up	1.62	Epdr1	BC014708	ependymin related protein 1 (zebrafish)
10429573	11.85	11.15	0.00105	up	1.63	Ly6c	BC010764|D86232	lymphocyte antigen 6 complex, locus C1 | lymphocyte antigen 6 complex, locus C2 | similar to Lymphocyte antigen 6C precursor (Ly-6C)
10595981	10.00	9.30	0.00137	up	1.63	Mras	AB004879	muscle and microspikes RAS
10442643	8.90	8.19	0.01104	up	1.63	Nme3	BC028503	non-metastatic cells 3, protein expressed in
10352314	7.53	6.82	0.01464	up	1.63	Lefty1	BC050221	left right determination factor 1
10504763	8.33	7.62	0.00388	up	1.64	Galnt12	BC056425	UDP-N-acetyl-alpha-D-galactosamine:polypeptide N-acetylgalactosaminyltransferase 12
10596747	8.65	7.92	1.52E-04	up	1.66	Sema3f	BC010976	sema domain, immunoglobulin domain (Ig), short basic domain, secreted, (semaphorin) 3F
10587639	7.76	7.02	0.00916	up	1.67	Nt5e	BC119268	5' nucleotidase, ecto
10412298	8.85	8.11	0.00456	up	1.67	Itga1	BC167237	integrin alpha 1
10497253	7.90	7.15	0.00499	up	1.67	Pmp2	BC099520	peripheral myelin protein 2
10392815	7.94	7.20	0.00159	up	1.67	AF251705|Cd300a	AF251705|AF251704	cDNA sequence AF251705 | CD300A antigen

10362538	11.09	10.35	0.0066	up	1.68	Lama4	U59865	laminin, alpha 4
10551966	10.77	10.01	9.77E-04	up	1.70	Hspb6	BC089621	heat shock protein, alpha-crystallin-related, B6
10358476	7.92	7.15	0.00752	up	1.71	Prg4	AB034730	proteoglycan 4 (megakaryocyte stimulating factor, articular superficial zone protein)
10437210	8.55	7.78	0.00755	up	1.71	Bace2	BC120773	beta-site APP-cleaving enzyme 2
10571860	9.20	8.43	1.68E-04	up	1.72	Hand2	AK035160	heart and neural crest derivatives expressed transcript 2
10407350	10.27	9.49	0.00476	up	1.72	Fgf10	BC048229	fibroblast growth factor 10
10427125	10.88	10.09	0.00682	up	1.73	Igfbp6	BC012723	insulin-like growth factor binding protein 6
10365974	11.52	10.72	0.00767	up	1.74	Dcn	BC132521	decorin
10458828	10.77	9.97	0.01321	up	1.74	Cdo1	BC013638	cysteine dioxygenase 1, cytosolic
10410766	8.69	7.89	0.01382	up	1.74	Nr2f1	BC108408	nuclear receptor subfamily 2, group F, member 1
10349773	9.75	8.94	0.00456	up	1.75	Klhdc8a	BC011135	kelch domain containing 8A
10436666	9.31	8.49	0.00976	up	1.76	Jam2	BC028778	junction adhesion molecule 2
10544875	10.64	9.82	0.00188	up	1.76	Scrn1	AK129084	secernin 1
10471067	11.59	10.78	0.00836	up	1.76	Prrx2	AK019971	paired related homeobox 2
10429568	11.72	10.90	4.15E-04	up	1.77	NM_010741	BC010764|M18466	lymphocyte antigen 6 complex, locus C1 | lymphocyte antigen 6 complex, locus C2 | similar to Lymphocyte antigen 6C precursor (Ly-6C)
10395849	7.62	6.80	0.00582	up	1.77	Pax9	BC005794	paired box gene 9
10399680	8.09	7.26	0.01509	up	1.77	Cys1	AF390548	cystin 1
10510129	11.32	10.50	0.00462	up	1.77	Dhrs3	BC010972	dehydrogenase/reductase (SDR family) member 3
10499132	10.21	9.38	0.00841	up	1.78	Mab21l2	AF126427	mab-21-like 2 (C. elegans)
10502805	8.22	7.38	6.41E-04	up	1.79	Ptgfr	D17433	prostaglandin F receptor
10370259	8.71	7.86	0.00304	up	1.79	Col18a1	BC064817	collagen, type XVIII, alpha 1
10365983	8.58	7.74	0.00905	up	1.79	Lum	BC005550	lumican
10563085	9.99	9.14	0.00144	up	1.80	Fcgrt	BC003786|AK179452	Fc receptor, IgG, alpha chain transporter
10590073	7.26	6.40	0.01516	up	1.80	Vill	AJ344341	villin-like
10382228	8.07	7.20	0.00894	up	1.82	Axin2	BC057338	axin2
10601848	10.69	9.82	0.00723	up	1.83	6530401D17Rik	BC016270	RIKEN cDNA 6530401D17 gene
10505008	8.57	7.69	0.00753	up	1.84	Slc44a1	AY249865	solute carrier family 44, member 1
10349174	8.70	7.81	0.00716	up	1.85	Serpinb8	BC025121	serine (or cysteine) peptdiase inhibitor, clade B, member 8
10492355	8.30	7.41	0.01218	up	1.86	Mme	BC034092	membrane metallo endopeptidase
10557058	9.79	8.89	0.00823	up	1.87	Polr3e	AK005034	polymerase (RNA) III (DNA directed) polypeptide E
10493798	10.97	10.06	1.02E-04	up	1.87	S100a16	BC020031	S100 calcium binding protein A16
10513061	9.80	8.90	8.98E-04	up	1.87	Ctnnal1	BC050070	catenin (cadherin associated protein), alpha-like 1
10380571	8.17	7.26	0.01195	up	1.87	Gngt2	BC061005	guanine nucleotide binding protein (G protein), gamma transducing activity polypeptide 2
10408557	9.23	8.33	0.00177	up	1.88	Serpinb1a	BC104333	serine (or cysteine) peptidase inhibitor, clade B, member 1a
10518967	9.70	8.79	6.39E-04	up	1.88	1190007F08Rik	BC099522|BC055936	RIKEN cDNA 1190007F08 gene
10490551	9.66	8.74	0.01333	up	1.89	Nkain4	BC089330	Na+/K+ transporting ATPase interacting 4
10474129	7.36	6.44	0.01315	up	1.89	Pamr1	BC031841	RIKEN cDNA E430002G05 gene
10585794	8.00	7.07	0.0075	up	1.90	Cyp11a1	AF195119	cytochrome P450, family 11, subfamily a, polypeptide 1
10490384	8.82	7.89	0.00375	up	1.90	Lama5	U37501	laminin, alpha 5
10563597	7.16	6.24	0.01009	up	1.90	Saa3	BC055885	serum amyloid A 3
10421258	9.24	8.30	0.00297	up	1.92	Pdlim2	BC024556	PDZ and LIM domain 2
10540523	8.14	7.19	1.18E-04	up	1.93	Lmcd1	BC019124	LIM and cysteine-rich domains 1
10496438	9.42	8.46	0.006	up	1.93	Adh1	BC013477	alcohol dehydrogenase 1 (class I)
10492169	8.68	7.73	0.0078	up	1.94	Mab21l1	AF228913	mab-21-like 1 (C. elegans)
10463716	8.88	7.92	0.0056	up	1.94	Cnnm2	AF216961	cyclin M2
10452648	9.41	8.44	0.00108	up	1.97	Emilin2	BC053753	elastin microfibril interfacer 2
10399465	9.18	8.18	4.03E-04	up	1.99	4a|4731402F03Rik|2310003N	BC005488	
10523120	7.93	6.94	0.00651	up	2.00	Cxcl5	BC024392	chemokine (C-X-C motif) ligand 5
10478415	10.43	9.41	0.01126	up	2.03	Wisp2	BC032877	WNT1 inducible signaling pathway protein 2
10571567	9.46	8.44	0.008	up	2.03	Sorbs2	BC039163	sorbin and SH3 domain containing 2
10596200	7.42	6.40	2.31E-04	up	2.03	Tmem108	BC052085	transmembrane protein 108
10607738	8.20	7.16	0.01279	up	2.05	Car5b	BC034413	carbonic anhydrase 5b, mitochondrial
10436978	10.60	9.56	0.0041	up	2.06	Cbr3	BC028763	carbonyl reductase 3
10603151	9.71	8.67	0.00536	up	2.06	Gpm6b	AF254875	glycoprotein m6b
10435961	8.23	7.18	0.0049	up	2.07	Gm10808	AK136967	
10423654	8.13	7.07	0.00376	up	2.08	Osr2	AF370121	odd-skipped related 2 (Drosophila)
10581664	8.35	7.27	5.13E-04	up	2.10	Il34	BC016254	RIKEN cDNA 2010004A03 gene
10572130	8.71	7.63	0.00109	up	2.11	Lpl	BC003305	lipoprotein lipase
10586118	8.98	7.91	0.00128	up	2.11	Calml4	AY061807	calmodulin-like 4
10374777	11.75	10.66	0.00364	up	2.12	Efemp1	AY251056	epidermal growth factor-containing fibulin-like extracellular matrix protein 1
10409314	10.00	8.92	5.08E-04	up	2.13	Msx2	AK133846	homeobox, msh-like 2 | similar to homeobox protein
10440099	10.15	9.06	0.00677	up	2.13	St3gal6	AF119390	ST3 beta-galactoside alpha-2,3-sialyltransferase 6
10445325	9.88	8.79	0.00259	up	2.13	Rcan2	BC049096	regulator of calcineurin 2
10384223	10.69	9.59	0.00161	up	2.14	Igfbp3	BC058261	insulin-like growth factor binding protein 3
10561128	7.28	6.18	0.00442	up	2.14	Cyp2s1	BC064733	cytochrome P450, family 2, subfamily s, polypeptide 1
10475532	10.87	9.77	0.00208	up	2.15	Sqrdl	AF174535	sulfide quinone reductase-like (yeast)
10503241	9.50	8.39	0.0092	up	2.17	Gdf6	AJ537425	growth differentiation factor 6
10416057	9.57	8.45	9.57E-04	up	2.17	Clu	D14077	clusterin
10594044	8.92	7.79	0.00509	up	2.18	Islr	BC006602	immunoglobulin superfamily containing leucine-rich repeat
10542470	10.55	9.41	0.00283	up	2.19	Mgst1	BC009155	microsomal glutathione S-transferase 1
10531931	9.08	7.94	0.00153	up	2.20	Sparcl1	U77330	SPARC-like 1 (mast9, hevin)
10404422	9.46	8.31	1.44E-05	up	2.21	Serpinb6b	U96707	serine (or cysteine) peptidase inhibitor, clade B, member 6b

10496425	10.14	9.00	7.94E-05	up	2.21	Adh7	AF331802	alcohol dehydrogenase 7 (class IV), mu or sigma polypeptide
10478048	7.47	6.32	0.01285	up	2.22	Lbp	BC004795	lipopolysaccharide binding protein
10469066	7.62	6.46	0.00602	up	2.22	Ccdc3	BC058616	coiled-coil domain containing 3
10542066	8.26	7.11	9.96E-04	up	2.23	Tspan11	BC057639	tetraspanin 11
10463476	10.02	8.85	2.15E-04	up	2.24	Kazald1	BC115642	Kazal-type serine peptidase inhibitor domain 1
10366476	8.84	7.67	8.87E-04	up	2.26	Ptprb	AY077755	protein tyrosine phosphatase, receptor type, B
10380250	8.18	6.99	0.00402	up	2.29	NR_003492	AL646096	RIKEN cDNA 2210409E12 gene
10516490	9.49	8.28	0.0031	up	2.30	Gjb5	M91236	gap junction protein, beta 5
10349947	11.51	10.30	0.00786	up	2.32	Fmod	BC064779	fibromodulin
10418300	8.41	7.19	0.01496	up	2.33	Cacna2d3	BC148700	calcium channel, voltage-dependent, alpha2/delta subunit 3
10521824	8.27	7.04	0.00676	up	2.35	Sod3	U38261	superoxide dismutase 3, extracellular
10593418	9.62	8.37	3.09E-04	up	2.38	Hspb2	BC064051	heat shock protein 2
10544891	7.68	6.42	0.00952	up	2.39	Nod1	BC042670	nucleotide-binding oligomerization domain containing 1
10352905	8.07	6.81	0.00178	up	2.39	Cd34	BC006607	CD34 antigen
10584883	10.26	8.99	0.00149	up	2.43	Fxyd6	BC051127	FXYD domain-containing ion transport regulator 6
10399555	7.76	6.47	0.00448	up	2.45	Kcnf1	BC053089	potassium voltage-gated channel, subfamily F, member 1
10506883	9.07	7.77	0.00512	up	2.46	Rab3b	BC051918	RAB3B, member RAS oncogene family
10453394	8.89	7.58	0.00447	up	2.46	Six2	BC068021	sine oculis-related homeobox 2 homolog (Drosophila) | similar to Homeobox protein SIX2 (Sine oculis homeobox homolog 2)
10414065	11.85	10.54	0.00979	up	2.49	Anxa8	BC030407	annexin A8
10574572	7.34	5.99	6.52E-04	up	2.55	2210023G05Rik	BC027185|BC024548	RIKEN cDNA 2210023G05 gene
10566034	9.52	8.17	3.86E-04	up	2.56	Folr1	AF096319	folate receptor 1 (adult)
10575844	9.46	8.07	0.00438	up	2.62	Cdh13	BC021628	cadherin 13
10511363	8.74	7.35	0.00745	up	2.62	Penk1	M55181	preproenkephalin 1
10361887	11.73	10.33	0.00341	up	2.63	Perp	AF249870	PERP, TP53 apoptosis effector
10413609	10.95	9.54	1.94E-04	up	2.65	Mustn1	BC116939|AJ277212	musculoskeletal, embryonic nuclear protein 1
10575693	7.81	6.39	0.00221	up	2.67	Vat1l	BC056927	
10547740	7.99	6.57	5.61E-04	up	2.67	C1s	BC018319	complement component 1, s subcomponent
10484463	10.36	8.94	1.54E-04	up	2.68	Serping1	AF010254	serine (or cysteine) peptidase inhibitor, clade G, member 1
10362959	8.46	7.04	0.01227	up	2.68	Popdc3	BC114993	popeye domain containing 3
10591169	7.81	6.37	0.00989	up	2.71	Muc16|LOC385009	AK003577	mucin 16
10393936	9.52	8.05	0.00301	up	2.77	Cbr2	BC010758	carbonyl reductase 2
10585194	9.28	7.79	0.00111	up	2.81	Il18	D49949	interleukin 18
10439500	12.34	10.83	0.01017	up	2.84	Upk1b	BC046604	uroplakin 1B
10404982	9.20	7.67	0.00113	up	2.90	Barx1	BC119047	BarH-like homeobox 1
10372145	10.06	8.52	8.85E-05	up	2.91	Alx1	BC052200	ALX homeobox 1
10540897	9.74	8.20	0.0035	up	2.92	Pparg	U01841	peroxisome proliferator activated receptor gamma
10485955	9.80	8.24	0.01529	up	2.93	Scg5	BC029021	secretogranin V
10357870	10.31	8.75	0.00863	up	2.96	Prelp	BC019775	proline arginine-rich end leucine-rich repeat
10427052	9.87	8.26	0.01108	up	3.05	Krt7	BC010337	keratin 7
10472809	9.02	7.41	0.00106	up	3.05	Dlx1	U51001	distal-less homeobox 1
10419566	8.98	7.34	0.00652	up	3.12	Ang2	BC146501	angiogenin, ribonuclease A family, member 2
10345065	8.68	7.02	0.00143	up	3.17	Gsta3	M73483	glutathione S-transferase, alpha 3
10565204	8.67	6.96	0.00139	up	3.25	Bnc1	U88064	basonuclin 1
10426767	9.91	8.16	1.06E-04	up	3.38	Aqp5	AF087654	aquaporin 5
10347277	12.92	11.13	9.50E-04	up	3.46	Igfbp2	BC012724	insulin-like growth factor binding protein 2
10424662	8.07	6.21	0.00225	up	3.63	Psca	AF319173	prostate stem cell antigen
10345921	10.64	8.77	0.00332	up	3.66	1500015O10Rik	BC002254	RIKEN cDNA 1500015O10 gene
10414025	8.86	6.95	0.01032	up	3.76	Gdf10	BC058358	growth differentiation factor 10
10381096	11.01	9.03	5.89E-05	up	3.92	Igfbp4	BC019836	insulin-like growth factor binding protein 4
10498584	9.62	7.64	0.00104	up	3.95	Rarres1	AK077393	retinoic acid receptor responder (tazarotene induced) 1
10538459	10.10	8.07	1.88E-04	up	4.07	Aqp1	L02914	aquaporin 1
10502961	9.30	7.13	1.62E-06	up	4.51	Lhx8	BC125281	LIM homeobox protein 8
10395389	7.95	5.77	0.0052	up	4.53	Sostdc1	AY255635	sclerostin domain containing 1
10380248	8.75	6.54	0.00295	up	4.62	G0000001305|OTTMUSG0000(	BC099439	
10461979	10.90	8.68	0.00291	up	4.66	Aldh1a1	BC054386	aldehyde dehydrogenase family 1, subfamily A1
10449000	10.07	7.81	1.58E-04	up	4.78	Msln	BC023753	mesothelin
10362495	10.51	8.24	0.00759	up	4.80	Col10a1	BC156192	collagen, type X, alpha 1
10571815	9.73	7.47	0.00702	up	4.81	Gpm6a	BC029711	glycoprotein m6a
10526441	10.19	7.91	3.67E-05	up	4.83	Upk3b	AY233463	uroplakin 3B
10354229	9.38	7.09	0.00295	up	4.89	2610017I09Rik	058417|BC049671|BC094	RIKEN cDNA 2610017I09 gene
10366446	9.59	7.07	0.01159	up	5.74	Tspan8	BC029808	tetraspanin 8
10345016	9.08	6.46	4.94E-04	up	6.13	Tcfap2b	AK017373	transcription factor AP-2 beta
10489463	9.97	7.26	0.00117	up	6.52	Slpi	U73004	secretory leukocyte peptidase inhibitor
10529651	8.65	5.59	0.00312	up	8.35	Msx1	BC016426	homeobox, msh-like 1
