# Supplementary figures and images for: Alkbh8 Regulates Selenocysteine-Protein Expression to Protect against Reactive Oxygen Species Damage
Source: PLoS One. 2015 Jul 6;10(7):e0131335. doi: 10.1371/journal.pone.0131335 (PMC4492958; doi:10.1371/journal.pone.0131335)

Supplemental Figure 1

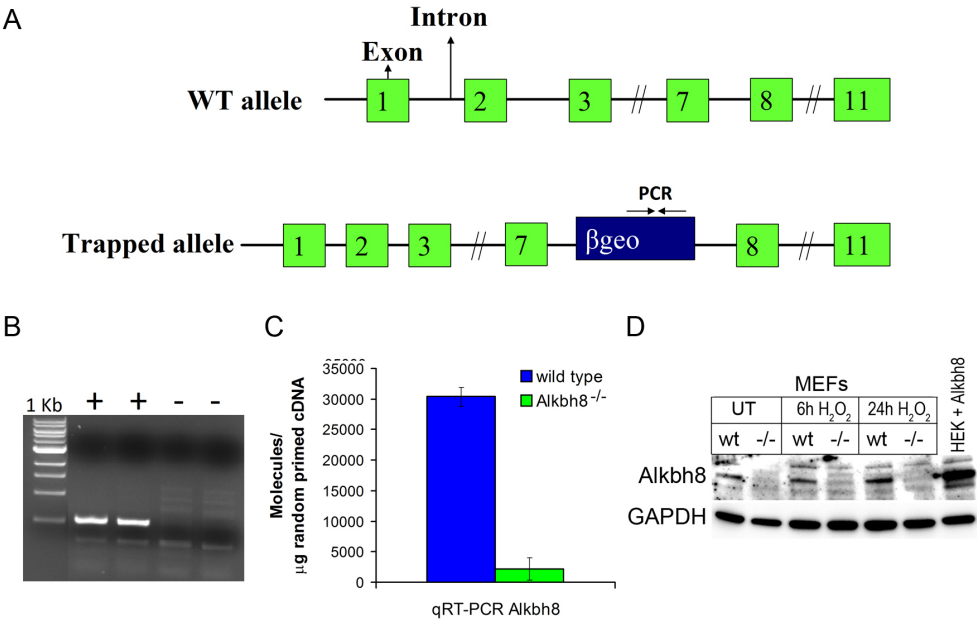

Supplement: S1 Fig — A) Embryonic stem cells with a gene trapped Alkbh8 allele, designated Alkbh8Gt(RRY122)Byg, were obtained from BayGenomics and used for injection into C57BL/6 blastocysts for the creation of Alkbh8-deficient heterozygous mouse [34]. Specific ß-galactosidase/neomycin resistance (ßgeo) vector insertion was mapped to chromosome 9:3335231–3385847 and occurs within intron 7 of the mouse Alkbh8 gene. B) PCR amplification with ßgeo specific primer sequences confirmed the presence of the ßgeo cassette in the ES cells. (C) Alkbh8 expression in MEFs was analyzed by qRT-PCR to establish gene copy number (D) and western blotting to confirm Alkbh8 protein expression in wt but not Alkbh8 -/- MEFs that were immortalized by sequential passage. Human embryonic kidney cells that over-expressed a full-length murine Alkbh8 expression plasmid (HEK + Alkbh8) served as a positive control. (PDF) [file pone.0131335.s002.pdf]

Supplemental Figure 2

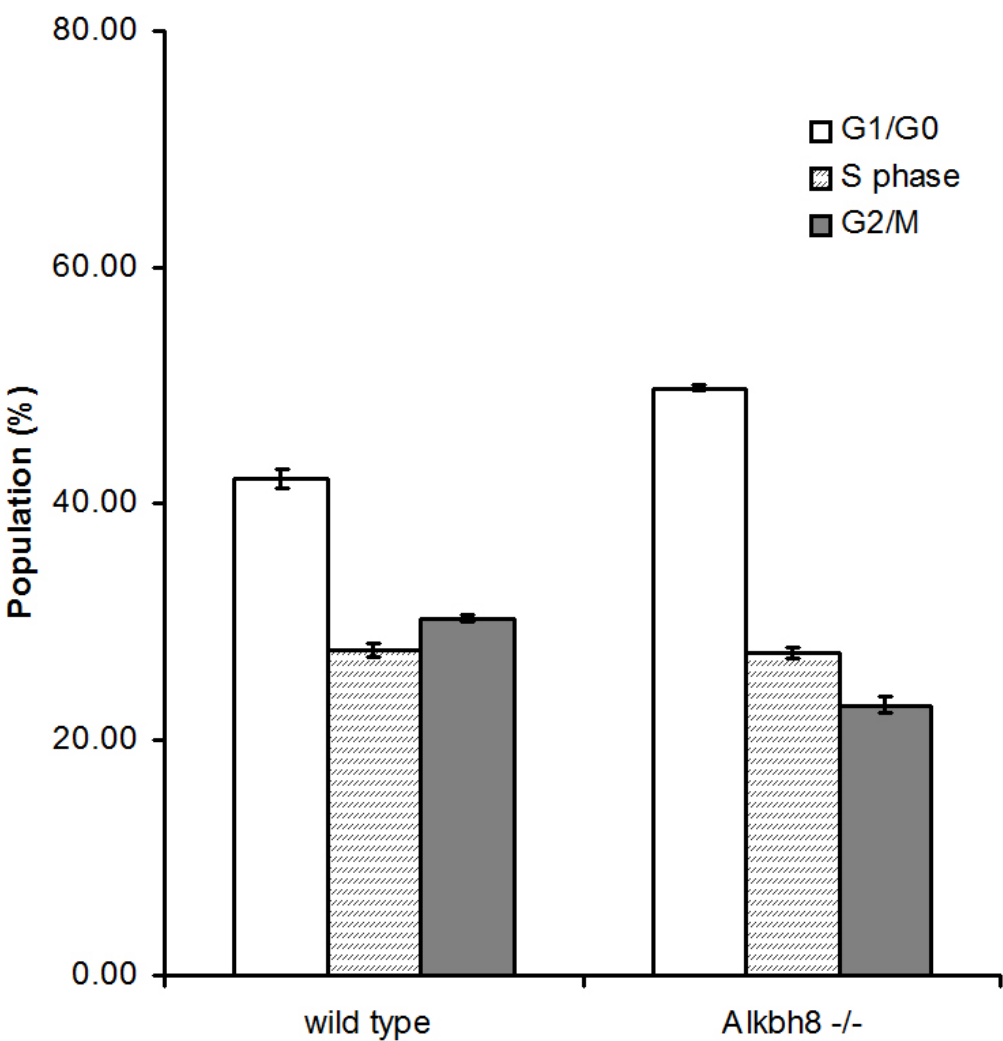

Supplement: S2 Fig — MEFs were stained with propidium iodide and the percentage of cells in G1/G0, S or G2/M phases of the cell cycle was determined by flow cytometry analysis of DNA content (±STDV, n = 3). (PDF) [file pone.0131335.s003.pdf]

Supplemental Figure 3

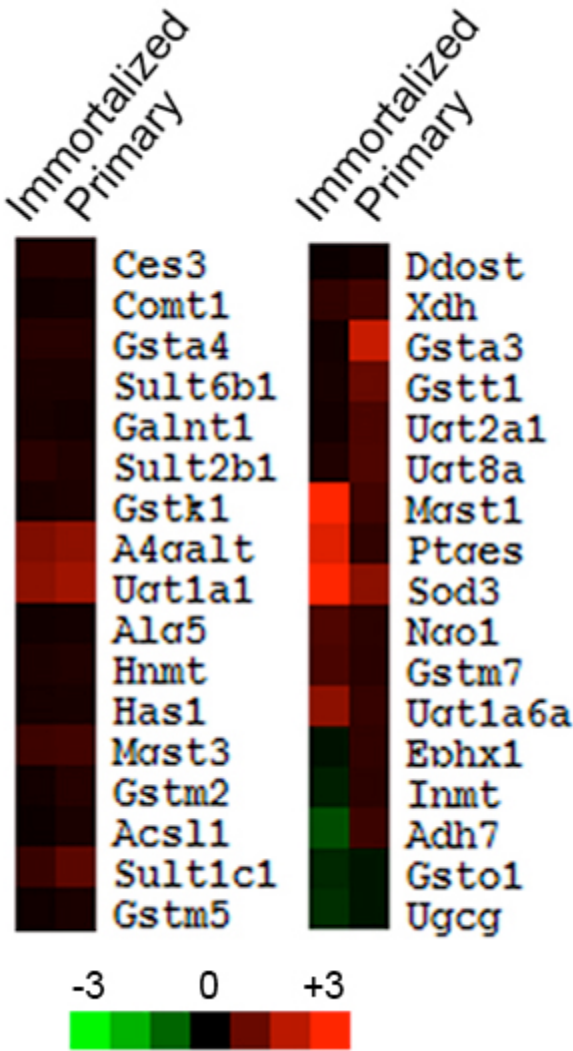

Supplement: S3 Fig — Microarray gene targets were confirmed as up-regulated targets by qRT-PCR gene expression analysis using RT2 Profiler (Qiagen) arrays of gene targets involved in the oxidative stress response. (PDF) [file pone.0131335.s004.pdf]

Supplemental Figure 4

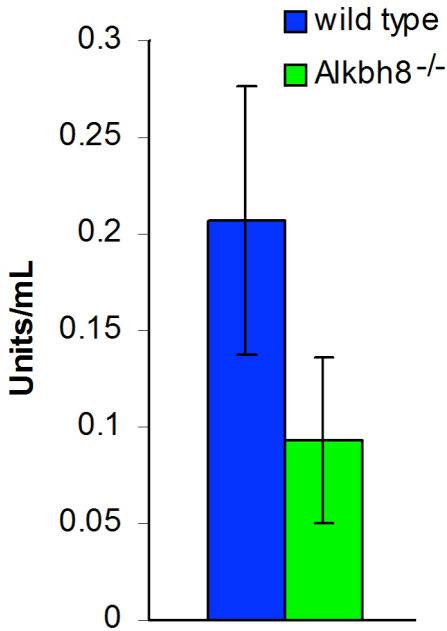

Supplement: S4 Fig — Gpx activity was measured in whole cell lysates prepared from wt and Alkbh8 -/- MEFs under basal growth conditions. (PDF) [file pone.0131335.s005.pdf]

Supplemental Figure 5

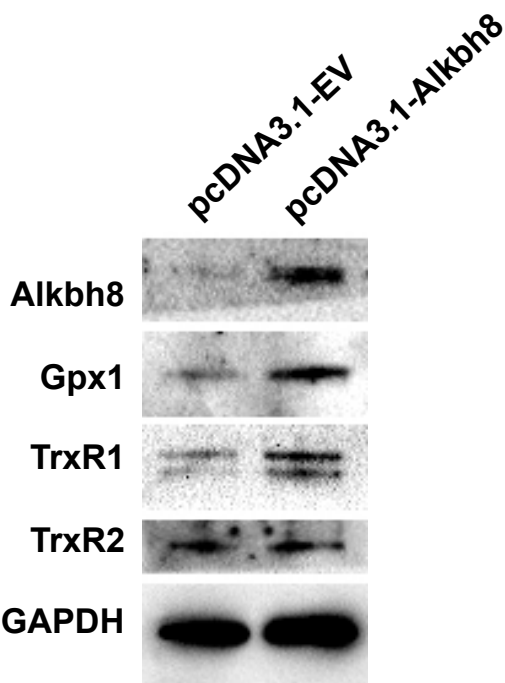

Supplement: S5 Fig — Alkbh8 -/- MEFs were necleofected with empty vector or an Alkbh8 expression vector. Immunoblot analysis was performed as described in Fig 5. (PDF) [file pone.0131335.s006.pdf]

Supplemental Figure 6

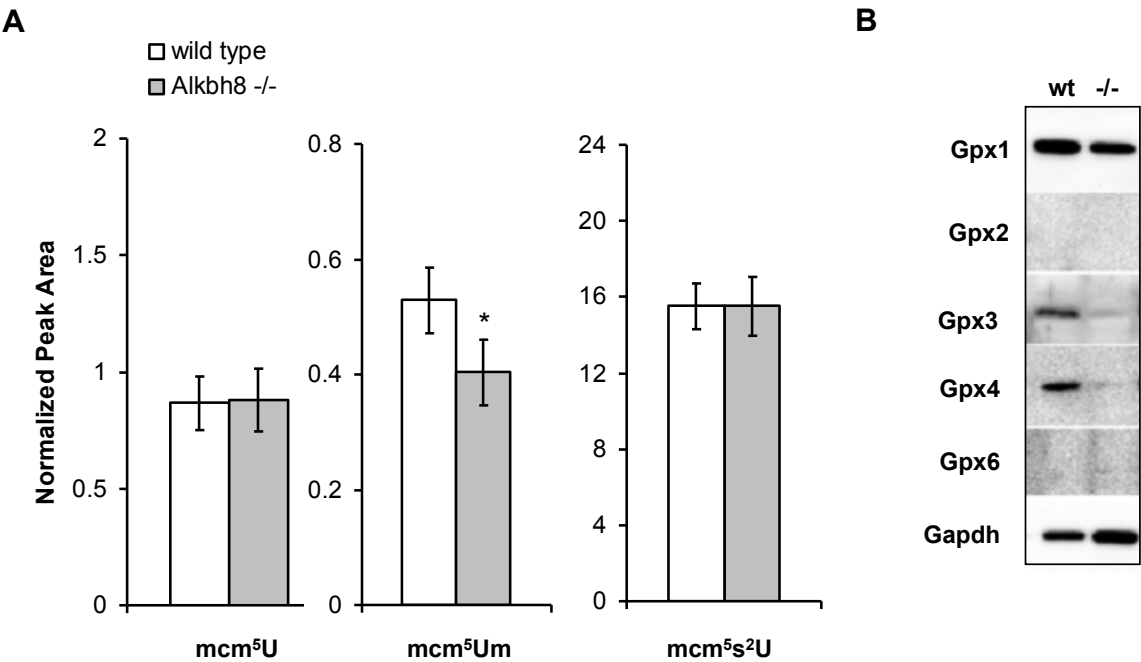

Supplement: S6 Fig — Protein and tRNA fractions were isolated from the livers of littermate-matched wild type and Alkbh8 -/- mice. (A) Mcm5-uridine based modifications were measured (N = 4) from the livers of wt and Alkbh8 -/- mice by HPLC-coupled mass spectrometry and quantified by integrating the normalized peak area intensity for each signal at the indicated post-exposure time points. Significant differences in mcm5Um modifications was determined by the Student’s t-test. (B) Gpx and Gapdh protein levels were analyzed in mouse liver extracts by immunoblots. (PDF) [file pone.0131335.s007.pdf]
